# Complement diagnostics and therapeutics for the pediatric population: early successes and opportunities for further advancement

**DOI:** 10.3389/fphar.2026.1891349

**Published:** 2026-09-10

**Authors:** Russell S. Whelan, Bradley P. Dixon

**Affiliations:** 1 Renal Section, Department of Pediatrics, University of Colorado School of Medicine, Aurora, CO, United States; 2 Department of Nephrology, Children’s Hospital Colorado, Aurora, CO, United States

**Keywords:** aHUS (atypical haemolytic uraemic syndrome), complement, complement diagnostics, complement pathway, complement therapeutics, health disparities, novel therapeutic agents, pediatric kidney disease

## Abstract

Complement-mediated diseases are individually rare, often rapidly progressive, and historically associated with high rates of permanent organ injury or death but are now among the most therapeutically actionable conditions in pediatric nephrology, hematology, and rheumatology. As demonstrated by thrombotic microangiopathy, and hemolytic uremic syndrome in particular, diagnostic delay can contribute directly to irreversible kidney injury. Complement inhibition has substantially improved outcomes in these conditions that previously carried a substantial risk of end-stage kidney disease. This review addresses the complement system as a clinical and therapeutic framework for the practicing pediatric clinician. The pathophysiology of the major complement-mediated diseases affecting children is examined, with atypical hemolytic uremic syndrome and transplant-associated thrombotic microangiopathy as central disease models alongside paroxysmal nocturnal hemoglobinuria, C3 glomerulopathy, IgA nephropathy, and ANCA-associated vasculitis. Approved complement inhibitors are reviewed by mechanistic position in the cascade, with attention to the clinical implications of terminal versus proximal pathway inhibition. Laboratory diagnosis, monitoring, and assessment of complement blockade adequacy are addressed, including practical limitations of complement testing in clinical practice. Critical gaps are examined, including diagnostic delays, absent validated biomarkers, inadequate pediatric trial infrastructure, and conditions without targeted therapies. Emerging therapeutics, point-of-care diagnostics, and pediatric-specific considerations including transition of care and reproductive counseling complete the review. Complement therapeutics have meaningfully altered the trajectory of some of the most severe and inadequately treated pediatric diseases. This field continues to evolve rapidly, with expanding indications and novel agents, though drug development challenges, diagnostic and monitoring gaps, and equitable consideration of pediatric application of these novel agents remain essential priorities.

## Section 1: complement biology and pediatric disease context

### Introduction

In pediatric medicine, complement-mediated diseases represent individually rare and rapidly progressive diseases that carry a high risk of permanent organ injury or death if diagnosis is delayed. Fortunately, recent therapeutic advances now allow meaningful interventions for conditions across nephrology, hematology, and rheumatology. The expansion of complement-targeted therapeutics over the past two decades has made working knowledge of complement biology directly relevant to clinical practice. This review provides an integrated framework linking complement pathway biology to disease-specific diagnosis, therapeutic selection, laboratory monitoring, and the identification of pediatric-specific gaps, organized around the needs of the practicing pediatric clinician. The conditions discussed are organized by the maturity of complement-targeted therapeutic evidence.

Pediatric complement-mediated disease presents challenges distinct from adult medicine. Genetic etiologies are more prevalent; complement components are present at reduced levels at birth (ranging 10%–80% of adult values, with even greater immaturity in premature newborns), introducing age-specific diagnostic complexity (McGreal et al., 2012). Organ injury during critical development windows carries consequences distinct from those in adults, with these children facing disease burden and treatment durations that may span several decades, affecting many for the entirety of their lives.

### The complement cascade

**FIGURE 1:**
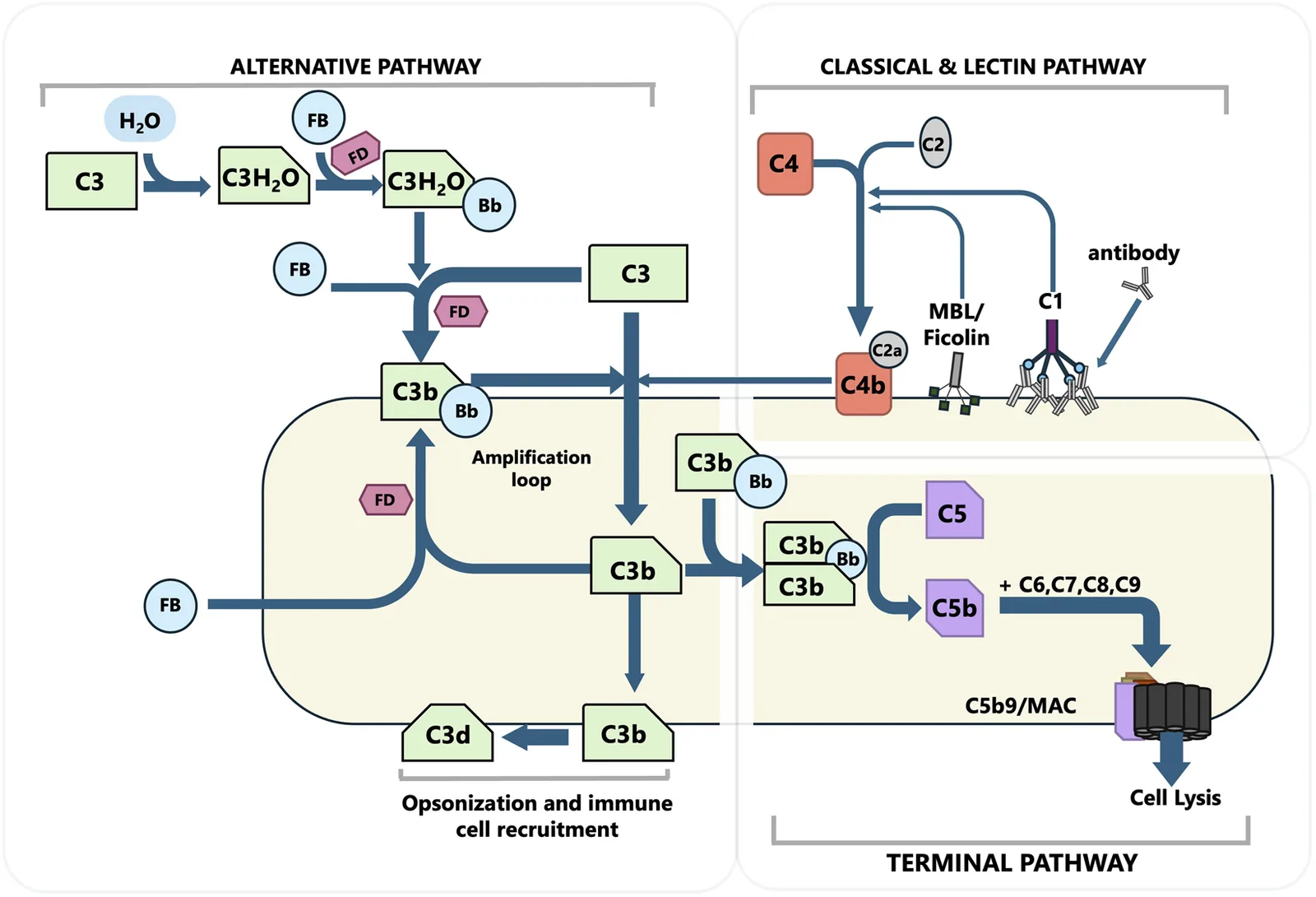
The complement cascade. The three activation pathways of the complement system and their convergence on common effector mechanisms are illustrated. In the alternative pathway (left), spontaneous hydrolysis of C3 generates C3(H_2_O), which associates with Factor B (FB); Factor D (FD) cleaves Factor B to produce the fluid-phase C3 convertase C3(H_2_O)Bb. Surface-deposited C3b similarly recruits Factor B and Factor D to form the amplification loop convertase C3bBb, driving progressive C3b deposition on unprotected surfaces. In the classical and lectin pathways (right), antibody-C1 complexes or mannose-binding lectin (MBL) recognition of surface patterns activates C4 and C2 to form the C3 convertase C4b2a. All pathways converge on C3 cleavage, generating C3b for opsonization and immune cell recruitment (bottom left) and C5 convertase assembly. C5 cleavage produces C5b, which initiates sequential recruitment of C6, C7, C8, and multiple C9 molecules to form the membrane attack complex (C5b-9/MAC), resulting in cell lysis (bottom right). FB, Factor B; FD, Factor D; MBL, mannose-binding lectin; MAC, membrane attack complex.

The complement system activates through three distinct pathways (classical, lectin, and alternative), converging on common effector mechanisms (Figure 1). The classical pathway is initiated by antigen-antibody complexes. C1q binds clustered immunoglobulins, activating associated serine proteases C1r and C1s that cleave C4 and C2 to form the classical pathway C3 convertase (C4b2a) (Merle et al., 2015). Classical pathway activation underlies antibody-mediated diseases including autoimmune hemolytic anemia, lupus nephritis, and antibody-mediated transplant rejection. The lectin pathway activates through molecular pattern recognition rather than antibody binding. Mannose-binding lectin (MBL) and ficolins recognize carbohydrate patterns on microbial surfaces, activating MBL-associated serine proteases (MASPs) that generate the same C3 convertase as the classical pathway (Dobo et al., 2024), providing immediate antibody-independent defense particularly important in early childhood. Low serum C4 on routine testing most commonly represents classical pathway consumption but is also seen with lectin pathway activation.

The alternative pathway is distinct from the classical and lectin pathways in that it does not require specific recognition molecules. Continuous low-level spontaneous hydrolysis of C3 (“tick-over”) generates a fluid-phase C3 convertase C3bBb that deposits C3b on nearby surfaces; on unprotected surfaces C3b persists and forms additional convertases, creating a powerful amplification loop. In experimental models, up to 80% of total complement effector generation has been attributed to alternative pathway amplification regardless of the initiating trigger (Harboe and Mollnes, 2008), meaning alternative pathway dysregulation can substantially worsen injury even in diseases initiated through other pathways.

All pathways converge at C3 cleavage, generating C3a (an anaphylatoxin) and C3b (an opsonin). C3b associates with existing convertases to form C5 convertases, which cleave C5 into C5a (the most potent anaphylatoxin) and C5b, initiating assembly of the membrane attack complex (MAC, C5b-9). The MAC forms transmembrane pores causing cell lysis; sublytic MAC deposition triggers cellular activation and inflammation, a mechanism increasingly recognized in complement-mediated kidney diseases (Takano et al., 2013). The position of a therapeutic target within this cascade determines its clinical consequences.

### Complement regulation

Regulatory mechanisms protect host tissues through fluid-phase and membrane-bound controls. Fluid-phase regulators include Factor H, the primary alternative pathway regulator, which binds C3b and acts as cofactor for Factor I-mediated C3b inactivation; Factor H dysfunction through mutation or autoantibody is the most common abnormality in pediatric aHUS (Michael et al., 2022). Factor I cleaves C3b and C4b into inactive fragments, requiring Factor H or membrane cofactor protein (MCP/CD46) as cofactors. The 5 Factor H-related proteins (FHRs), encoded in the same chromosomal region as CFH, compete with Factor H for C3b binding—an interaction that can both modulate Factor H activity and, in the absence of normal FHR balance, amplify alternative pathway activation. Deletions of *CFHR* genes, particularly *CFHR1*, underlie autoantibody-mediated aHUS (DEAP-HUS). C1 inhibitor (C1-INH) inactivates C1r, C1s, and MASPs, regulating classical and lectin pathway initiation. C4 binding protein (C4BP) serves as a cofactor for Factor I, and accelerates C3 convertase decay in the fluid phase.

Membrane-bound regulators provide cell-surface protection: MCP serves as a cofactor for Factor I; decay-accelerating factor (DAF/CD55) accelerates C3 convertase decay; CD59 prevents MAC assembly. Loss of CD55 and CD59 in PNH renders blood cells susceptible to complement-mediated destruction, and CD55 deficiency alone causes CHAPLE syndrome.

### Pediatric complement-mediated diseases

#### Thrombotic microangiopathy and hemolytic uremic syndrome

Thrombotic microangiopathy (TMA) is characterized by microangiopathic hemolytic anemia, thrombocytopenia, and end-organ injury from endothelial damage and microvascular thrombosis. The clinical triad of microangiopathic hemolysis, thrombocytopenia, and acute kidney injury defines hemolytic uremic syndrome (HUS), the prototypical TMA in children.

Thrombotic thrombocytopenic purpura (TTP), caused by severe ADAMTS13 deficiency (<10% activity), should be excluded early in TMA evaluation, as it requires plasma exchange rather than complement inhibition. TTP is rare in children compared with adults; congenital TTP (Upshaw-Schulman syndrome) is the predominant pediatric form due to genetic deficiency of the ADAMTS13 protein, typically presenting in the neonatal period or early childhood. ADAMTS13 activity should be sent at presentation alongside initial complement studies.

Shiga toxin-producing *Escherichia coli* HUS (STEC-HUS) accounts for approximately 90% of pediatric HUS and presents with a bloody diarrheal prodrome followed by the classic triad. STEC-HUS is primarily toxin-mediated (Shiga toxin causes direct endothelial injury and platelet activation), and management is supportive. Complement activation markers are elevated during acute disease and complement regulatory gene variants may modulate disease severity in susceptible individuals (Fremeaux-Bacchi et al., 2019) but complement-targeted therapy has not unequivocally proven efficacy in this disease (Ives et al., 2024; Garnier et al., 2023), and its role in severe or refractory cases remains investigational.

#### Atypical HUS

Atypical HUS (aHUS) results from dysregulated alternative pathway activation on endothelial surfaces and represents the paradigmatic complement-mediated TMA (Loirat and Fremeaux-Bacchi, 2011). Loss-of-function mutations limiting the function of complement regulatory proteins on the endothelial surface allow unchecked alternative pathway amplification. Such mutations occur in approximately 60% of pediatric cases; the most commonly affected genes include Factor H (*CFH*, 20%–30%), membrane cofactor protein (*MCP/CD46*, 10%–15%), Factor I (*CFI*, 5%–10%), and thrombomodulin (*THBD*, 3%–5%) (Loirat and Fremeaux-Bacchi, 2011). Conversely, gain-of-function mutations in *C3* (5%–10%) and Factor B (*CFB*, 1%–3%) drive overactive complement activation.

Acquired aHUS also results from anti-Factor H autoantibodies, identified in approximately 10% of pediatric cases (substantially higher in some global settings including India) (Puraswani et al., 2019) and strongly associated with homozygous deletions of *CFHR1* (DEAP-HUS) (Skerka et al., 2009). Loss of these FHR proteins likely permits autoantibody development through impaired immune tolerance, and recognition of DEAP-HUS is therapeutically important as these patients may benefit from plasma exchange and immunosuppression in addition to complement inhibition.

Serum C3 is reduced in only 30%–50% of patients; a normal C3 does not exclude aHUS, as complement activation may be restricted to endothelial surfaces (Loirat and Fremeaux-Bacchi, 2011). Prior to complement inhibition, up to 50% of children with aHUS died or reached end-stage kidney disease within the first year (Loirat and Fremeaux-Bacchi, 2011). Complement inhibition has transformed outcomes, and aHUS represents one of the most significant successes in complement therapeutics. Kidney transplantation in the aHUS patient requires particular attention: children with mutations affecting circulating proteins (Factor H, Factor I, C3, Factor B) carry recurrence risk exceeding 80% without prophylaxis, as the allograft’s endothelium remains exposed to the same dysregulated systemic complement (Loirat and Fremeaux-Bacchi, 2011). Prophylactic complement inhibition has made transplantation viable for these children. While most soluble complement proteins are generated by the liver, kidney transplantation for aHUS patients with *MCP* mutations carry low recurrence risk, as the allograft does not express mutated MCP on its cell surfaces.

*DGKE* mutations (diacylglycerol kinase epsilon deficiency) cause a TMA phenotype that presents almost exclusively in the first year of life and can mimic complement-mediated aHUS but operates through a complement-independent mechanism. Acute TMA episodes tend to recur in the first 5 years of life and then diminish, but persistent proteinuria and progressive chronic kidney disease are the long-term phenotype; 80% of patients remain free of end-stage kidney disease at 10 years, though chronic proteinuria is nearly universal (Brocklebank et al., 2020; Azukaitis et al., 2017). Critically, DGKE-associated HUS does not respond to complement inhibition, with relapses reported to occur despite adequate C5 blockade. Establishing an appropriate diagnosis avoids unnecessary and ineffective therapy in these patients. No targeted treatment exists, representing an ongoing therapeutic gap.

Cobalamin C deficiency (methylmalonic aciduria with homocystinuria) due to biallelic mutations in the *MMACHC* gene, represents another non-complement form of TMA presenting in neonates and infants that requires metabolic rather than complement-directed management.

#### Transplant-associated thrombotic microangiopathy

TA-TMA complicates 10%–30% of pediatric hematopoietic stem cell transplants and is at least partially driven by lectin pathway-mediated endothelial injury in the transplant conditioning environment. Clinical features overlap substantially with graft-versus-host disease and calcineurin inhibitor toxicity, making diagnosis challenging. International consensus criteria incorporating soluble C5b-9 (sC5b-9) measurement provide both diagnostic and risk-stratification thresholds, with elevated sC5b-9 central to both diagnosis and prognostication (Schoettler et al., 2023; Schoettler et al., 2025a).

#### Paroxysmal nocturnal hemoglobinuria (PNH)

PNH results from somatic *PIGA* mutations in hematopoietic stem cells, causing absence of GPI-anchored complement regulators CD55 and CD59 and rendering red blood cells susceptible to complement-mediated lysis (Hill et al., 2017). Pediatric cases present with hemolytic anemia, hemoglobinuria, fatigue, and potentially life-threatening thrombosis; cytopenias from underlying bone marrow failure are common, and PNH may coexist with aplastic anemia (Sun and Babushok, 2020). Terminal complement inhibition dramatically reduces intravascular hemolysis and thrombotic risk, though C3 fragments continue depositing on PNH erythrocytes despite terminal pathway blockade. This may lead to extravascular hemolysis through hepatosplenic clearance of C3b-opsonized cells in approximately 20%–25% of patients, which has led to adjunctive therapeutic development of proximal complement inhibitors.

#### Cold agglutinin disease (CAD)

CAD in children is most often infection-associated, with *Mycoplasma* pneumoniae and Epstein-Barr virus as the principal triggers. IgM autoantibodies activate the classical pathway at cool peripheral temperatures, generating C3b-mediated hemolysis; the direct antiglobulin test is positive for C3d but negative for IgG, distinguishing CAD from warm autoimmune hemolytic anemia. Pediatric CAD rarely requires complement-targeted therapy; evidence for complement inhibition in this indication derives from adult trials, most notably the phase 3 CADENZA trial of sutimlimab (Roth et al., 2022).

#### C3 glomerulopathy

C3 glomerulopathy (C3G) encompasses glomerular diseases defined by predominant C3 deposition with minimal immunoglobulin staining, reflecting fluid-phase alternative pathway dysregulation (Pickering et al., 2013; Smith et al., 2019). The two subtypes are dense deposit disease (DDD) and C3 glomerulonephritis (C3GN), and both share uncontrolled alternative pathway C3 convertase activity as their common mechanism. Approximately 50%–80% of patients harbor nephritic factors (C3 nephritic factor or C5 nephritic factor) that stabilize their respective convertases, and 20%–30% carry mutations in *CFH*, *CFI*, *MCP*, *C3*, or *CFB* (Servais et al., 2012; Osborne et al., 2018) or rearrangements in the *CFH-CFHR* gene locus. Serum C3 is depressed in 50%–70% at presentation; persistent C3 depression beyond 8–12 weeks after an apparent infection-triggered nephritis should prompt biopsy to exclude C3G (Osborne et al., 2018). Prognosis is guarded, with 30%–50% reaching end-stage kidney disease within 10 years, and complement-targeted therapy is now approved for this indication. Immune-complex membranoproliferative glomerulonephritis (IC-MPGN) shares the membranoproliferative pattern seen in C3G biopsies but is distinguished by significant immunoglobulin co-deposition. Secondary causes such as infection, autoimmune disease, and monoclonal gammopathy must be excluded, though drivers of complement dysregulation may be identified in a significant proportion of cases, particularly in children, and there is increasingly recognized overlap in the therapeutic management of C3G and IC-MPGN.

#### IgA nephropathy

IgA Nephropathy (IgAN) is the most common chronic glomerulonephritis worldwide and frequently presents in children with episodic gross hematuria accompanying upper respiratory infections. Although traditionally classified as an immune complex disease, aberrantly glycosylated IgA1 triggers complement activation through the lectin and alternative pathways, driving progressive mesangial C3 accumulation, and complement deposition intensity correlates with histologic severity (Maillard et al., 2015). Complement-targeted therapy is now approved for IgAN in adults, with pediatric use under active investigation (NCT06994845 and NCT07024563).

#### ANCA-associated vasculitis

ANCA-associated vasculitis (AAV) involves alternative pathway amplification of neutrophil-mediated injury: ANCA activation of neutrophils triggers complement, generating C5a that primes further neutrophil activation through C5aR1 in a feed-forward loop (Garred et al., 2021). Selective C5a receptor blockade was reported to reduce glucocorticoid exposure in adults, although the pivotal trial has since been retracted (see Section 2).

#### Additional complement-mediated conditions

Several other conditions merit recognition. In acetylcholine receptor antibody-positive myasthenia gravis, complement activation at the neuromuscular junction drives MAC-mediated motor endplate destruction; complement inhibition is effective in refractory disease (Howard, 2018). CHAPLE syndrome, caused by CD55 loss-of-function mutations, produces severe protein-losing enteropathy through uncontrolled intestinal complement activation, with terminal complement inhibition producing marked clinical improvement in reported cases, though the evidence base remains limited to small cohorts (Ozen et al., 2017; Ozen, 2019). CD59 deficiency causes MAC-mediated hemolytic anemia and chronic inflammatory demyelinating polyneuropathy (CIDP), presenting predominantly in childhood (Klemann et al., 2018). In systemic lupus erythematosus, genetic C1q deficiency predisposes to disease through impaired apoptotic debris clearance, while complement activation drives immune complex-mediated organ injury once disease is established; childhood-onset lupus nephritis carries a more aggressive phenotype with higher rates of renal involvement and increased mortality compared with adult-onset disease (Coss et al., 2023; Amaral et al., 2014). Hereditary angioedema (HAE) can occur from C1-INH deficiency, but HAE attacks are bradykinin-mediated, and management targets the kallikrein-kinin system rather than complement directly (Sinnathamby et al., 2023).

## Section 2: current therapeutics for complement-mediated disease

### Introduction

The complement therapeutic landscape encompasses agents acting at each major node along the cascade: classical pathway inhibitors that block the initiating C1 complex, lectin pathway inhibitors that selectively target MASP-2, terminal pathway inhibitors that prevent C5 cleavage and thereby block both C5a generation and MAC (C5b-9) assembly, proximal inhibitors that arrest the cascade at C3 or at the alternative pathway convertase components Factor B and Factor D, and C5a receptor antagonists that selectively block inflammatory C5a signaling while leaving MAC formation intact (Mastellos et al., 2024; Zelek et al., 2019).

The distinction between proximal and terminal complement inhibition carries direct clinical implications that recur throughout this review. Terminal inhibitors leave C3-mediated opsonization intact; proximal inhibitors provide more complete cascade blockade at the cost of eliminating such opsonization.

### Non-complement-specific immunomodulation

Conventional immunosuppressants such as corticosteroids, mycophenolate, and calcineurin inhibitors do not directly modulate complement activation, but instead modulate complement-stimulated inflammation. These agents lack pathway specificity and carry substantial short- and long-term toxicity, limiting their benefit in pediatric treatment of complement-mediated diseases. While plasma exchange served as the standard of care in acute aHUS before eculizumab availability, its contemporary role is limited to bridging therapy when urgent complement inhibition is needed, or in the setting of DEAP-HUS to remove the offending autoantibody. Despite prior use in complement-mediated disease, IVIG has no established complement-specific mechanism (Arnson et al., 2009).

Addressing upstream autoimmune or inflammatory triggers may be mechanistically appropriate in select cases but may not directly resolve complement dysregulation. Rituximab or cyclophosphamide are often utilized in anti-Factor H antibody-positive aHUS to specifically target autoantibody generation (Sana et al., 2014). In C3G, rituximab may blunt autoantibody production but does not directly block alternative pathway activation and its benefit as monotherapy is inconclusive (Rousset-Rouviere et al., 2014). Despite these examples of adjunctive therapy, complement-focused therapies remain the ideal choice in effective management of complement-mediated disease.

### Targeted complement blockade

Targeted complement inhibitors are organized below by their position in the cascade, from terminal pathway agents acting at C5 through classical and lectin pathway inhibitors to proximal agents targeting C3 and the alternative pathway amplification loop. C5a receptor antagonism, which intercepts inflammatory signaling downstream of C5 cleavage while leaving MAC formation intact, is addressed last given its distinct mechanistic position.

### Terminal complement inhibitors

**FIGURE 2:**
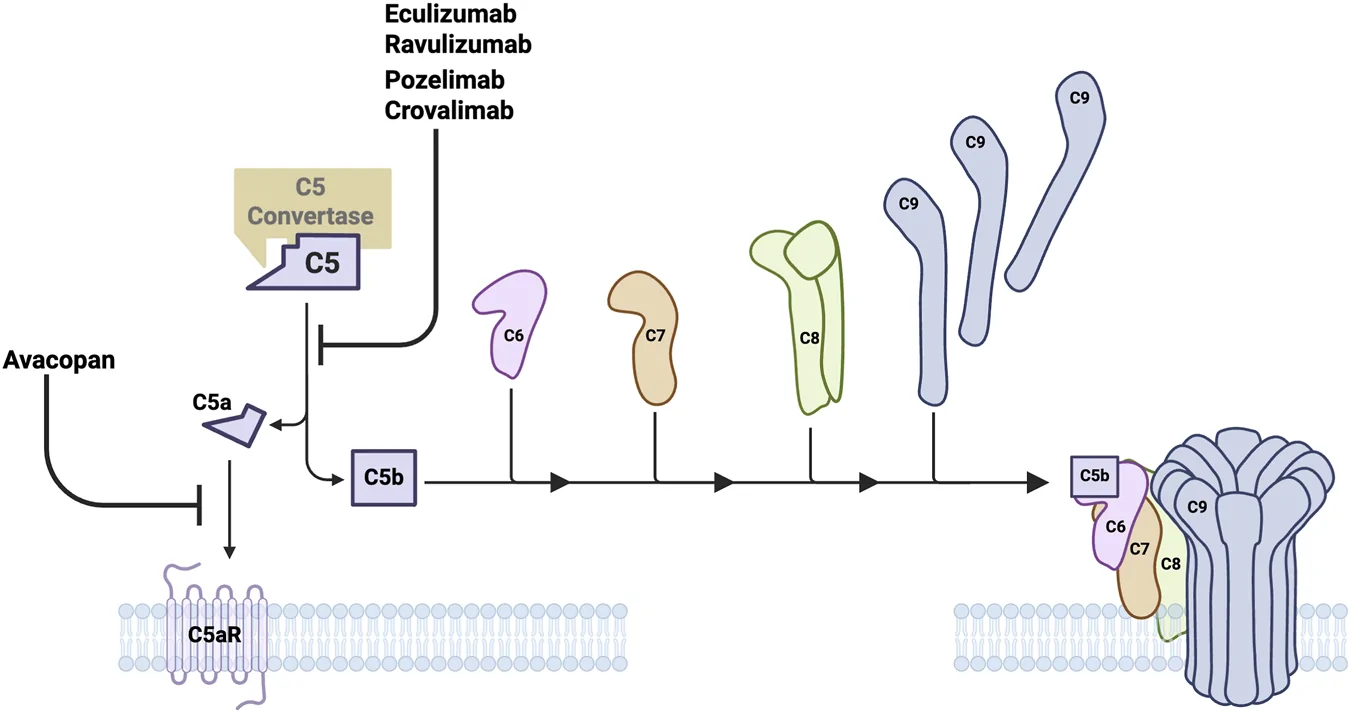
Therapeutic targets in the terminal complement pathway. Approved agents acting at and downstream of C5 cleavage are shown at their respective sites of action. Anti-C5 monoclonal antibodies—eculizumab, ravulizumab, pozelimab, and crovalimab—prevent C5 cleavage, blocking both C5a-mediated inflammatory signaling and membrane attack complex assembly. Avacopan selectively blocks C5a receptor signaling downstream of C5 cleavage while leaving MAC assembly intact. C5aR, C5a receptor; MAC, membrane attack complex.

Terminal pathway inhibitors established proof of concept for targeted complement therapeutics by demonstrating that selective blockade could safely and effectively treat previously lethal diseases (Figure 2) (Patriquin and Kuo, 2019). Susceptibility to encapsulated bacteria—particularly *Neisseria* species—remains the defining infectious risk of this therapeutic class and applies to all agents that suppress terminal pathway activity (Garred et al., 2021).

#### Eculizumab (Soliris)

Eculizumab, a humanized monoclonal antibody that binds C5 and prevents its cleavage by C5 convertases, received its first FDA approval in 2007 for paroxysmal nocturnal hemoglobinuria (Patriquin and Kuo, 2019), transforming a disease associated with severe hemolysis, thrombosis, and early mortality into a manageable chronic condition (Gavriilaki et al., 2022). A critical limitation is that C3 fragments continue to deposit on PNH erythrocytes despite C5 blockade, causing extravascular hemolysis through hepatosplenic clearance of C3b-opsonized cells in approximately 20%–25% of patients—a finding that drove development of proximal complement inhibitors discussed below (Duval et al., 2026).

The 2011 approval for atypical hemolytic uremic syndrome represented eculizumab’s most significant pediatric impact (Michael et al., 2022; Gurevich and Landau, 2023). Eculizumab produces hematologic normalization within days to weeks in most patients, with improvement or stabilization of kidney function for most patients. Children treated promptly may achieve complete renal recovery, while delayed treatment (e.g., aHUS initially misdiagnosed as STEC-HUS) may result in residual impairment (Antonucci et al., 2024; Sellier-Leclerc et al., 2007; Fakhouri et al., 2014). Prevention of post-transplant recurrence with appropriate complement inhibition transformed aHUS from a near-contraindication to transplantation into a condition where outcomes approach those of other kidney diseases.

Although not FDA-approved for TA-TMA, eculizumab has become the primary complement-directed treatment for high-risk TA-TMA at many centers experienced in this complication, supported by observational and prospective cohort data rather than randomized trial evidence. In the first prospective multi-institutional study in children and young adults with high-risk TA-TMA and multi-organ dysfunction, eculizumab therapy was associated with 6-month and 1-year survival of 71% and 62% respectively, compared with 16.7% in historical untreated controls with the same high-risk features. Dosing guided by eculizumab drug levels and sC5b-9 monitoring was integral to the protocol (Jodele et al., 2024). Earlier retrospective data from the same group demonstrated comparable survival improvement in 64 pediatric patients (Jodele et al., 2020).

Eculizumab is approved for pediatric aHUS at all ages including neonates, with weight-based induction dosing (weekly for 4 weeks by weight category) followed by every-two-week maintenance; younger children demonstrate faster drug clearance and may require dose intensification, and therapeutic drug monitoring can guide individualized dosing when available (Gurevich and Landau, 2023).

Eculizumab is additionally FDA-approved for refractory generalized myasthenia gravis and neuromyelitis optica spectrum disorder, with limited pediatric experience in both indications (Howard et al., 2017; Pittock et al., 2019). Limited studies investigating eculizumab treatment for CD59 deficiency have demonstrated improvements in both hemolytic anemia and CIDP (Almutawea et al., 2023), with encouraging Phase 2a clinical trial results (Mevorach et al., 2016), although larger studies remain to be performed.

#### Ravulizumab (Ultomiris)

Ravulizumab is a second-generation C5 inhibitor engineered with pH-dependent binding to its C5 target and Fc region modification enhancing neonatal Fc receptor (FcRn) binding, extending the elimination half-life approximately four-fold to 48–52 days and enabling every-eight-week maintenance dosing in older children and adults (Ricklin, 2024). Non-inferiority to eculizumab in PNH was established in the ALXN1210-PNH-301 and -302 trials, with superior pharmacokinetic stability and fewer breakthrough hemolysis episodes, and received FDA approval for PNH in adults in 2018, with the pediatric indication following in 2021. Ravulizumab received FDA approval for pediatric and adult aHUS in 2019 (Gavriilaki et al., 2022) following positive clinical trial outcomes for adult (Rondeau et al., 2020) and pediatric (Ariceta et al., 2021) patients. Ravulizumab is approved in children with weight-based dosing; children weighing 5 to less than 20 kg require every-four-week maintenance due to faster clearance, while those 20 kg or more follow adult-equivalent intervals (Gurevich and Landau, 2023). The reduction from biweekly to every-eight-week infusions meaningfully reduces treatment burden over a typically lifelong treatment course (Dixon and Sabus, 2022; Mauch et al., 2023).

### Breakthrough complement activation with C5 inhibitors

Breakthrough complement activation can occur during infections, surgery, trauma, or other highly inflammatory states despite ongoing C5 inhibitor therapy, manifesting as recurrent hemolysis in PNH or recurrent TMA in aHUS (Gavriilaki and Brodsky, 2020). Two mechanisms underlie breakthrough events. Pharmacokinetic breakthrough results when complement activation intensity exceeds available drug, with management involving dose intensification or interval shortening, with these approaches guided by free C5 levels when available. Pharmacodynamic breakthrough may also reflect incomplete C5 inhibition despite adequate drug levels, most commonly due to C5 polymorphisms that reduce inhibitor binding affinity; the c.2654G>A (p.Arg885His) variant abrogates eculizumab and ravulizumab binding. Recognition of this polymorphism has driven development of C5 inhibitors with distinct epitopes, most notably crovalimab, which binds the C5 beta chain and is therefore unaffected by alpha chain polymorphisms (Nishimura et al., 2014).

#### Pozelimab (Veopoz)

Pozelimab is a fully human IgG4 anti-C5 monoclonal antibody approved by the FDA in August 2023 for CHAPLE syndrome (CD55 deficiency with hyperactivation of complement, angiopathic thrombosis, and protein-losing enteropathy) (West et al., 2024; Ozen et al., 2024). CD55 loss-of-function mutations eliminate decay-accelerating factor function on intestinal vascular endothelium, producing protein-losing enteropathy, hypoalbuminemia, growth failure, and recurrent thrombosis. Pozelimab is unique among complement therapeutics in offering subcutaneous weekly maintenance administration approved for patients 1 year of age and older, with demonstrated efficacy including rapid resolution of protein-losing enteropathy, albumin normalization, and thrombosis prevention—a particularly important profile for young children with limited venous access.

#### Crovalimab (Piasky)

Crovalimab is a humanized anti-C5 monoclonal antibody approved in June 2024 for PNH in patients aged 13 years and older weighing at least 40 kg. Crovalimab binds a distinct epitope on the C5 beta chain, unaffected by the p.Arg885His polymorphism that abrogates eculizumab and ravulizumab binding to C5. In addition to the same FcRn-enhancing mutations shared with ravulizumab, crovalimab also incorporates superior pH-dependent C5 binding, maintaining high-affinity binding at physiologic pH while dissociating rapidly in the acidic endosomal environment for enhanced antibody recycling. Furthermore, its IgG1 backbone was engineered to eliminate FcγR and C1q binding, reducing off-target complement and effector function activation. These targeted modifications collectively improve C5 neutralization efficiency and extend circulatory half-life, making crovalimab uniquely suited to subcutaneous administration (Sampei et al., 2024). Following intravenous loading and four weekly subcutaneous loading doses, maintenance is subcutaneous every 4 weeks, enabling home self-administration. Non-inferiority to eculizumab in treatment-naive PNH was demonstrated in the COMMODORE 2 trial (Roth et al., 2024). Clinical trial investigation of crovalimab in pediatric and adult aHUS is ongoing (NCT04958265, NCT04861259), and early pediatric TMA experience is accumulating, including a case series in pediatric HUS (Jiang et al., 2026) and a report of successful rescue in complement-mediated TMA refractory to ravulizumab (Aigner et al., 2026).

### Classical pathway inhibition

#### Sutimlimab (Enjaymo)

Sutimlimab is a humanized IgG4 monoclonal antibody targeting C1s that provides selective classical pathway blockade and was FDA-approved in 2022 for cold agglutinin disease in adults (Roth et al., 2021; Roth et al., 2022). By preventing antibody-triggered C1 complex activation, sutimlimab reduces hemolysis and transfusion requirements in CAD; dosing is intravenous every 2 weeks. Sutimlimab has not been approved for the pediatric population, and off-label use for pediatric CAD has not been reported in the literature to date.

### Lectin pathway inhibition

#### Narsoplimab (Yartemlea)

Narsoplimab is a fully human monoclonal antibody that selectively inhibits MASP-2, the effector enzyme of the lectin pathway, while preserving classical and alternative complement functions. The FDA approved narsoplimab in December 2025 for hematopoietic stem cell transplant-associated thrombotic microangiopathy (TA-TMA) in adults and children aged 2 years and older—the first approved therapy for TA-TMA and the first approved lectin pathway inhibitor. Approval was based on demonstration of favorable TMA response rates, with comparative analyses showing an associated survival advantage over supportive care (Matsui et al., 2026), in a condition where no prior standard therapy existed.

Narsoplimab is administered intravenously at weight-based doses once weekly, with a median treatment duration of 8 weeks in clinical studies. Notably, narsoplimab carries no boxed warning, requires no REMS program, and does not require meningococcal vaccination prior to treatment, reflecting preservation of terminal and alternative pathway function with selective lectin pathway inhibition. Its approval for children aged 2 years and older is particularly significant given the frequency and historically poor outcomes of TA-TMA in pediatric hematopoietic stem cell transplantation.

### Proximal complement inhibition: targeting C3 and the alternative pathway

**FIGURE 3:**
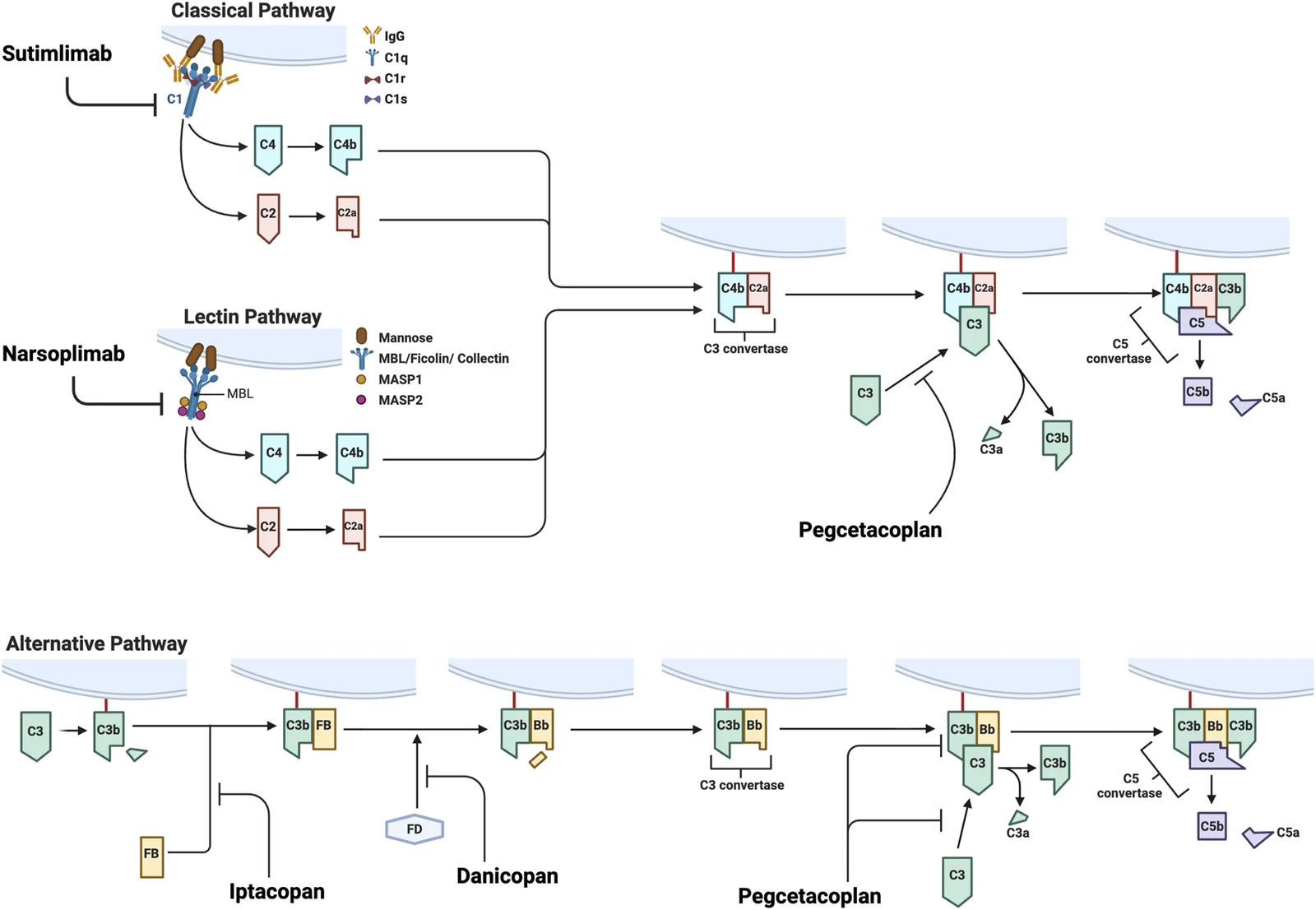
Therapeutic targets in the classical, lectin, and alternative pathways. Therapeutic targets in the classical, lectin, and alternative complement pathways are illustrated, with approved inhibitors shown at their respective sites of action. Upper panel: classical and lectin pathway inhibition at C1s, MASP-2, and C3. Lower panel: alternative pathway inhibition at Factor B, Factor D, and C3. FD, Factor D; FB, Factor B; MBL, mannose-binding lectin; MASP, MBL-associated serine protease.

The pathogenesis of C3G illustrates the mechanistic rationale for proximal inhibition most clearly (Figure 3). Fluid-phase alternative pathway dysregulation drives persistent C3 convertase activity and glomerular C3 fragment deposition. A substantial proportion of patients harbor C3 nephritic factors or C5 nephritic factors that stabilize their respective convertases (Servais et al., 2012; Ravindran et al., 2018; Marinozzi et al., 2017a). In patients with C3 nephritic factor activity, terminal pathway inhibition leaves upstream C3 convertase dysregulation unaddressed—underscoring why proximal inhibition is mechanistically better aligned to C3G pathophysiology. The trade-off across all proximal inhibitors is elimination of C3-mediated opsonization, raising more substantial concerns of infection with encapsulated organisms than those associated with terminal pathway blockade alone (Garred et al., 2021).

#### Pegcetacoplan (Empaveli/Aspaveli)

Pegcetacoplan is a pegylated bicyclic peptide that binds C3 and its activated fragments (C3b, iC3b), inhibiting all C3 convertases regardless of initiating pathway and blocking the cascade at its central amplification step (Lamers et al., 2022; Kolev et al., 2023). FDA approval for PNH came in 2021 based on the PEGASUS trial (Hillmen et al., 2021), which demonstrated pegcetacoplan’s superiority to eculizumab in improving hemoglobin levels and reducing transfusion requirements by controlling both intravascular and extravascular hemolysis. Administration is subcutaneous twice weekly via infusion pump.

Early experience across open label clinical trials and several case series exploring the use of pegcetacoplan in C3 glomerulopathy demonstrated proteinuria reduction and kidney function stabilization in children (Holle et al., 2018; Dixon et al., 2023; Mancuso et al., 2025; Roman Ortiz et al., 2026; Guzman and Perry, 2025; Alconcher et al., 2026). In July 2025, the FDA approved pegcetacoplan for C3 glomerulopathy and primary immune complex membranoproliferative glomerulonephritis based on the phase 3 VALIANT study (Fakhouri et al., 2025). This randomized, double-blind trial in patients age 12 and older demonstrated substantial proteinuria reduction with pegcetacoplan versus placebo, with a majority of treated patients achieving clinically meaningful proteinuria reduction and eGFR stabilization. The trial’s inclusion of adolescents from age 12 establishes safety and efficacy in this group, relevant given that pediatric C3G frequently follows an aggressive course with high rates of progression to end-stage kidney disease.

#### Iptacopan (Fabhalta)

Iptacopan is an oral, small-molecule Factor B inhibitor that selectively blocks alternative pathway C3 convertase formation, with FDA approval for PNH in December 2023. By preventing Factor B interaction with C3b and subsequent cleavage by Factor D, iptacopan specifically inhibits the alternative pathway. Similar to pegcetacoplan, iptacopan controls both intravascular and extravascular hemolysis by preventing C3 convertase generation. This drug represents the first orally bioavailable selective complement inhibitor in clinical use (Li and Woodruff, 2025), with oral twice-daily dosing offering meaningful convenience compared to parenteral C5 inhibitors.

Iptacopan received FDA accelerated approval for IgA nephropathy in adults in August 2024, with final 24-month data from the APPLAUSE-IgAN trial confirming significant slowing of kidney function decline and a meaningful reduction in composite kidney failure risk compared to placebo (Antonucci et al., 2024; Barratt et al., 2026). In March 2025, iptacopan received FDA approval for C3G in adults based on the APPEAR-C3G trial, which demonstrated proteinuria reduction with favorable eGFR effects (Kavanagh et al., 2025). Both approvals are currently limited to adults; extension to pediatric populations will require dedicated pharmacokinetic and efficacy studies currently in progress. Iptacopan is also being explored in aHUS both as induction treatment (APPLEHUS, NCT04889430) and in patients switching from terminal complement inhibition (APPRECIATE, NCT05935215).

#### Danicopan (Voydeya)

Danicopan is an oral, small-molecule Factor D inhibitor approved by the FDA in April 2024 as add-on therapy to eculizumab or ravulizumab for extravascular hemolysis in adults with PNH. By reversibly inhibiting Factor D, danicopan prevents C3b opsonization of PNH erythrocytes while patients continue terminal pathway blockade, addressing the subset of PNH patients with clinically significant extravascular hemolysis despite adequate C5 inhibition. The phase 3 ALPHA trial demonstrated significant hemoglobin improvement with the addition of danicopan to C5 inhibitor therapy compared to placebo (Lee et al., 2023). Danicopan is dosed orally three times daily, requires meningococcal vaccination and REMS enrollment, and warrants liver function monitoring given observed hepatic enzyme elevations. Pediatric studies and investigation as potential monotherapy are anticipated. Danicopan did not demonstrate comparable efficacy in reducing proteinuria or improving histological activity score in a clinical trial of adults with C3 glomerulopathy (Nester et al., 2022).

Three alternative pathway-targeted agents—pegcetacoplan, iptacopan, and danicopan—are now commercially available with different mechanisms, administration routes, and side effect profiles (Ricklin, 2024). Whether C3 inhibition offers advantages over Factor B or Factor D inhibition in specific disease contexts remains to be established; head-to-head comparative trials have not been conducted.

### C5a receptor antagonism

#### Avacopan (Tavneos)

Avacopan is an oral, small-molecule C5aR1 antagonist that blocks C5a signaling on neutrophils, monocytes, and other inflammatory cells while leaving C5 cleavage, C5b generation, and MAC assembly intact (Garred et al., 2021). This selective targeting of C5a signaling theoretically preserves MAC-mediated bacterial killing, though clinical data have not confirmed a meaningful reduction in infectious risk compared to complete C5 blockade.

The phase 3 ADVOCATE trial (Jayne et al., 2021) reported superior sustained remission and substantially reduced glucocorticoid exposure with avacopan versus a prednisone taper, leading to FDA approval in 2021 for adults with severe active AAV (granulomatosis with polyangiitis or microscopic polyangiitis). A post hoc analysis of the trial reported encouraging kidney outcomes in the subgroup with kidney involvement (Geetha et al., 2025). However, the primary ADVOCATE publication (Jayne et al., 2021) was retracted in June 2026 after an FDA investigation identified undisclosed postunblinding readjudication of primary endpoint assessments (Rubin, 2026). The FDA has proposed withdrawal of avacopan’s approval, citing data integrity and hepatotoxicity concerns, and the European Commission revoked its EU marketing authorization in August 2026, with US proceedings ongoing at the time of writing. Because the post hoc kidney analysis derives from the same trial, the efficacy evidence for avacopan must be interpreted with substantial caution. For pediatric AAV, where glucocorticoid toxicity profoundly impacts growth and development, C5aR1 antagonism remains mechanistically attractive, but application awaits resolution of these concerns. The phase 2 ACCOLADE trial in C3G did not meet its primary endpoint, with no significant difference in disease activity scores between avacopan and placebo after 26 weeks (Bomback et al., 2025). Liver function monitoring is required; hepatotoxicity signals identified by the FDA include severe drug-induced liver injury and vanishing bile duct syndrome.

### Practical considerations for initiating complement inhibition

The decision to initiate complement inhibition in pediatric patients requires careful consideration of indication, timing, vaccination status, and family preparedness for long-term therapy.

#### Infection prophylaxis

Complement inhibition substantially increases susceptibility to encapsulated bacterial infections, with meningococcal disease incidence increased approximately 1,000- to 2,000-fold compared to the general population in patients receiving C5 inhibitors (Garred et al., 2021). This risk is not theoretical: pediatric patients are particularly vulnerable given age-related immune immaturity, the frequency of daycare and school exposure, and incomplete primary vaccination series that may precede a complement-mediated disease diagnosis. All patients must receive meningococcal conjugate vaccine (MenACWY) and meningococcal serogroup B vaccine (MenB) before initiating therapy, ideally at least 2 weeks prior to allow protective immunity to develop. Children who have not completed age-appropriate primary meningococcal series should receive catch-up vaccination. Neonates and young infants requiring urgent complement inhibition present a particular challenge, as meningococcal vaccines cannot be administered before 2 months of age and require multiple doses to achieve protective immunity. In this population, antibiotic prophylaxis is the sole protective measure until vaccination can be completed, and close infectious disease surveillance is essential. When urgent treatment precludes waiting, as in acute aHUS, vaccination should be given immediately with concurrent antibiotic prophylaxis maintained for at least 2 weeks post-vaccination. Vaccination alone provides established but incomplete protection, as breakthrough meningococcal disease has been reported in vaccinated patients, including cases caused by nongroupable *Neisseria* meningitidis not covered by standard vaccines (McNamara et al., 2017); continuous penicillin V, amoxicillin, or azithromycin prophylaxis throughout therapy is therefore a reasonable and widely used strategy in pediatric practice, with agent-specific guidance available for complement inhibitors approved for kidney disease (Java et al., 2026). All patients and families should be counseled on the signs of meningococcal disease (fever, headache, stiff neck, rash).

#### Baseline laboratory studies

Essential baseline studies prior to initiating complement inhibition include: complete blood count with differential and reticulocyte count; comprehensive metabolic panel with creatinine, electrolytes, and liver function tests; lactate dehydrogenase, haptoglobin, and indirect bilirubin as hemolysis markers; complement studies including C3, C4, alternative pathway activity, and disease-specific assays such as sC5b-9 when available; and disease-specific markers including proteinuria quantification in kidney disease or PNH clone size by flow cytometry.

## Section 3: laboratory testing in complement-mediated disease

### Introduction

Unlike most clinical laboratory tests, complement assays are extremely sensitive to specimen handling, require pathway-specific interpretation, and are not standardized across laboratories—factors that make accurate diagnosis dependent on understanding not just which tests to order, but how and when to use them. Comprehensive evaluation integrates clinical presentation, standard and specialized complement assays, genetic analysis, and histopathology (Mastellos et al., 2024).

### Specimen collection and processing

Complement proteins and activation fragments are labile, and preanalytical error is a major source of spurious results (Ekdahl et al., 2018). Serum is used for complement protein levels and functional assays; EDTA plasma is required for complement activation markers (C3a, C5a, sC5b-9, Bb), as EDTA prevents *ex vivo* activation during handling. Samples must be processed within 1–2 h or frozen at −80 °C; freeze-thaw cycles must be avoided (Willrich et al., 2021a; Nandakumar et al., 2025). Age-specific reference ranges should be used, as neonates and young infants have lower complement levels than older children and adults.

### Diagnostic testing

#### Serum complement levels

C3 and C4 are offered by most clinical laboratories and serve as useful first-line screening tests (Willrich et al., 2021a). Low C3 with normal C4 suggests alternative pathway activation, as seen in aHUS, C3G, and some forms of post-infectious glomerulonephritis (PIGN). Low C3 and C4 together indicate classical pathway activation, as in lupus nephritis, cryoglobulinemia, and immune complex diseases. Normal C3 and C4 do not exclude complement-mediated disease (activation may be tissue-localized or balanced by hepatic synthesis), and serum C3 is reduced in only 30%–50% of aHUS patients (Ekdahl et al., 2018). Serum C5 is not routinely measured; during C5 inhibitor treatment, free C5 can be measured at select reference laboratories to assess blockade adequacy, though availability is very limited (Gavriilaki and Brodsky, 2020).

#### CH50 and AH50

CH50 and AH50 serve as essential first-tier screening tests for complement disorders ranging from recurrent infections to autoimmune diseases and thrombotic microangiopathies (Ekdahl et al., 2018; Willrich et al., 2021a). CH50 is widely available; AH50 varies by institution, and ELISA-based alternatives (e.g., Wieslab) offer advantages in sample volume and standardization over traditional hemolytic assays. Very low values for CH50 and/or AH50 warrant individual component evaluation; only CH50 is FDA-approved, and other functional assays are laboratory-developed tests subject to inter-laboratory variability (Nandakumar et al., 2025).

#### Factor H and factor I functional assays

Factor H and Factor I functional activity can be assessed through specialized assays measuring cofactor activity for C3b inactivation, useful when variants of uncertain significance are identified or clinical suspicion persists despite normal quantitative levels.

#### Complement activation markers

Complement activation markers (C3a, C3d, C5a, Bb, Ba, sC5b-9) require EDTA plasma and specialized processing; they provide direct evidence of *in vivo* activation and help distinguish deficiency from consumption (Ekdahl et al., 2018).

#### Soluble C5b-9 (sC5b-9)

sC5b-9 is the most extensively validated marker for monitoring complement inhibitor therapy (Ricklin et al., 2017; Mohebnasab et al., 2019). In active aHUS, if sC5b-9 is elevated, it generally normalizes with eculizumab (Wehling et al., 2017), correlating with remission, though levels may normalize despite ongoing disease in some patients. In transplant-associated TMA, sC5b-9 serves both diagnostic and prognostic roles: pretransplant median sC5b-9 is approximately 92 ng/mL (range 47–127 ng/mL), and levels at or above 244 ng/mL indicate active disease by consensus criteria—a threshold derived from clinical outcome data rather than population reference ranges, which vary by platform from approximately 200–300 ng/mL. High-risk TA-TMA, defined by both elevated sC5b-9 and proteinuria, carries a 1-year non-relapse mortality exceeding 80% without complement-directed therapy (Schoettler et al., 2023). sC5b-9 guides risk stratification and treatment response assessment in TA-TMA, with normalization below 244 ng/mL defining complete response by consensus criteria (Schoettler et al., 2025a). Interpretation during active C5 inhibitor therapy requires caution, as therapeutic antibodies can interfere with sC5b-9 measurements in some commercial assays (Willrich et al., 2021b). Urinary sC5b-9, reflecting intrarenal rather than filtered plasma activation (given its high molecular weight precluding glomerular filtration), has been explored as a biomarker of intrarenal complement activation but remains investigational.

#### C3 and factor B activation fragments

C3d measurement has particular utility in detecting C3d-opsonized erythrocytes in PNH patients with extravascular hemolysis (Gavriilaki et al., 2022). In C3G, persistently elevated C3d and low C3 despite eculizumab therapy reflect ongoing alternative pathway activation at the C3 level that terminal pathway inhibition cannot address (Wehling et al., 2017). Alternative pathway activation fragments Bb and Ba help distinguish pathway-specific activation patterns and are particularly useful in conditions where alternative pathway dysregulation is the primary driver (Ekdahl et al., 2018; Ricklin et al., 2017).

#### Assay standardization and limitations

Inter-laboratory variability is a recognized limitation in complement diagnostics: reference standards are lacking for most complement components and activation markers beyond C3 and C4, and results cannot be directly compared across laboratories until harmonized standards are established (Nandakumar et al., 2025; Ricklin et al., 2017; Prohaszka et al., 2016).

### Autoantibody testing

#### C3 nephritic factor

C3 nephritic factor (C3NeF) stabilizes the alternative pathway C3 convertase, prolonging its half-life and driving persistent C3 consumption. Published cohort data report C3NeF positivity in 40%–50% of C3G patients overall (Servais et al., 2012; Ravindran et al., 2018), with detection rates varying by cohort composition, histologic subtype, and assay methodology; C3NeF is more frequently detected in DDD than in C3GN, and its presence does not invariably correlate with disease activity. C3NeF testing is clinically available through reference laboratories and should be included in the initial evaluation of suspected C3G.

#### C4 and C5 nephritic factors

C4 nephritic factor (C4NeF) stabilizes the C4b2a convertase of the classical and lectin pathways and is identified in 5%–15% of C3G cases. C5 nephritic factor (C5NeF) stabilizes the C5 convertase, is detected in approximately 49% of patients when systematically tested and is more common in C3GN than DDD (Marinozzi et al., 2017a). These autoantibodies should be considered in patients with complement dysregulation without identifiable C3NeF; testing is available primarily through specialized research laboratories.

#### Anti-factor H and other autoantibodies

Anti-Factor H autoantibodies occur in approximately 10% of aHUS patients and 3% of C3G patients, with aHUS-associated antibodies targeting C-terminal domains, and C3G-associated antibodies targeting N-terminal regions (Zhang et al., 2020; Jozsi et al., 2021). Testing should be performed in all children presenting with aHUS, as their presence is strongly associated with acquired disease (DEAP-HUS), a distinct subtype of aHUS prompting the use of immunosuppression in addition to complement inhibition (Michael et al., 2022). Anti-Factor B and anti-C3b autoantibodies are also described in C3G, occurring in ∼3% of patients (Marinozzi et al., 2017b; Hauer et al., 2024); testing for these is available in specialized laboratories (Ricklin et al., 2017).

#### Genetic testing

Genetic testing is integral to evaluating complement-mediated diseases, particularly aHUS and C3G (Michael et al., 2022). Comprehensive panels typically include *CFH*, *CFI*, *CD46* (*MCP*), *C3*, *CFB*, *THBD*, *CFHR1–5*, and *DGKE*; next-generation sequencing allows simultaneous analysis with rapid turnaround. The mutation location within CFH has direct phenotypic implications: N-terminal mutations affecting fluid-phase regulation predispose to C3G, while C-terminal mutations affecting cell surface binding predispose to aHUS (Osborne et al., 2018). The *CFH/CFHR* region is prone to deletions, duplications, and hybrid gene formation due to high sequence homology; standard sequencing misses these copy number variants, and Multiplex Ligation-dependent Probe Amplification (MLPA) should be included in comprehensive genetic evaluation. C5 polymorphisms are also clinically relevant in the context of complement inhibitor selection: the p.Arg885His variant (c.2654G>A), present in approximately 3% of Japanese individuals (Nishimura et al., 2014) but rare in most other populations, abrogates eculizumab and ravulizumab binding and should be considered when evaluating patients with poor treatment response.

### Interpretation and counseling

Genetic testing aids in establishing diagnosis, informs prognosis, guides transplant planning, and enables family screening; interpretation requires expertise given the frequency of variants of uncertain significance and the incomplete penetrance characteristic of complement gene variants, and should include genetic counseling (Gurevich and Landau, 2023).

### Renal histopathology and complement staining

Kidney biopsy provides direct evidence of complement deposition and activation and is the primary histopathological examination in pediatric complement-mediated kidney diseases.

#### Immunofluorescence microscopy

Standard panels include immunoglobulins (IgG, IgA, IgM), complement (C3, C1q), and light chains (κ and λ). Staining patterns and intensity provide critical diagnostic information.

C3 immunofluorescence can detect glomerular C3 fragments (C3b, iC3b, C3c); C3d also is covalently bound to cell surfaces and persists after activation ceases, highlighting past rather than ongoing activity. C3 deposition on biopsy demonstrates complement involvement but does not specify pathway—alternative, classical, and lectin pathway activation all produce C3 deposition. C1q co-deposition raises suspicion for classical pathway involvement, though its absence does not exclude it. Properdin or Factor B staining, available only in specialized laboratories, can help identify alternative pathway-predominant disease (Osborne et al., 2018; Itami et al., 2020).

C3-dominant staining (C3 intensity at least two orders of magnitude greater than immunoglobulin) with minimal immunoglobulin defines C3 glomerulopathy (Vivarelli et al., 2022). The “full house” pattern (IgG, IgA, IgM, C3, C1q) characterizes lupus nephritis. Isolated or predominant IgA with C3 characterizes IgA nephropathy and IgA vasculitis.

C4d staining is important in transplant pathology, where peritubular capillary C4d deposition indicates antibody-mediated rejection (Naesens et al., 2024). C4d, a stable and covalently-bound footprint of classical or lectin pathway activation, is performed by immunofluorescence (frozen tissue, more sensitive) or immunohistochemistry (paraffin) and is routinely available in transplant pathology laboratories. C4d staining may also be performed in settings other than transplant pathology to evaluate glomerular diseases, with its positivity indicating classical or lectin pathway activation, although its use in these settings is not routine.

#### Electron microscopy

Electron microscopy provides ultrastructural detail classifying glomerulopathies, evaluating deposit morphology, location, and electron density. In C3G, deposit characteristics distinguish dense deposit disease—highly electron-dense, osmophilic, sausage- or ribbon-shaped intramembranous deposits within the glomerular basement membrane—from C3 glomerulonephritis, which shows subendothelial, mesangial, and subepithelial deposits of variable and less uniform density (Vivarelli et al., 2022). In thrombotic microangiopathies such as aHUS, electron microscopy reveals endothelial swelling, subendothelial expansion with electron-lucent material, and glomerular basement membrane changes characteristic of thrombotic microangiopathy.

### Monitoring and treatment response

#### General principles

Monitoring complement-targeted therapy serves several purposes: confirming treatment response, detecting relapse or breakthrough activation, assessing complement blockade adequacy, and identifying treatment complications (Antonucci et al., 2024; Mohebnasab et al., 2019). International consensus emphasizes individualized monitoring based on relapse risk, genotype, and clinical evolution (Michael et al., 2022); specific blockade adequacy targets are addressed in the relevant subsections below.

### Disease-specific monitoring

#### Hemolytic uremic syndrome and atypical hemolytic uremic syndrome

In acute presentations, priority specimens should be collected before complement inhibitor initiation, as treatment profoundly alters complement biomarker results. This minimum set includes C3, C4, CH50, sC5b-9, anti-Factor H antibodies, EDTA plasma for additional complement activation markers (i.e., C3a, C5a, Bb), and EDTA whole blood for genetic banking.

Monitoring thereafter focuses on the classic triad of microangiopathic hemolytic anemia, thrombocytopenia, and kidney function (Michael et al., 2022). Hematologic parameters include CBC with platelet count, LDH, haptoglobin, and peripheral smear for schistocytes. Platelet count is typically the earliest response marker, normalizing within days to weeks; LDH decline and haptoglobin recovery follow. During early maintenance, weekly to monthly monitoring for 6 months is recommended, with frequency thereafter guided by genotype and clinical stability in aHUS; monitoring duration and intensity in STEC-HUS is guided by clinical recovery, as the majority resolve without relapse.

Renal monitoring includes creatinine, eGFR, BUN, urinalysis, and urine protein quantitation; renal recovery typically lags hematologic improvement, and residual impairment reflects the extent of pre-treatment injury.

Circulating sC5b-9 normalizes with effective C5 inhibition and may serve as a pharmacodynamic endpoint, though it can remain normal despite ongoing endothelial injury in some patients. Serum-induced endothelial C5b-9 deposition, available at specialized centers, is more sensitive than circulating sC5b-9 for detecting residual complement activity at the target cell surface; normalization correlated with treatment response and guided dose optimization in clinical studies (Noris et al., 2014; Galbusera et al., 2019). Persistent low C3 during treatment may indicate ongoing upstream activation not captured by sC5b-9 alone. These sC5b-9 and C3 monitoring principles apply primarily to aHUS; in STEC-HUS, complement biomarker monitoring is not routinely required given the self-limited course in most patients.

Monitoring considerations differ by agent. Eculizumab requires every-two-week infusions with trough targets of 50–100 μg/mL for aHUS; children demonstrate faster drug clearance and frequently require dose intensification or interval shortening (Gurevich and Landau, 2023). Ravulizumab allows every 8-week dosing with a recommended trough target of greater than 175 μg/mL; its more consistent pharmacokinetic profile reduces the variability that can complicate aHUS management, though vigilance during intercurrent illness remains important given the extended dosing interval (Gavriilaki et al., 2022). The free C5 immunoassay used as the primary pharmacodynamic endpoint in ravulizumab trials (threshold <0.5 μg/mL for complete inhibition) represents a specialized test and is not currently available as a routine clinical laboratory test. Commercially available C5 assays measure functional activity or total C5 concentration rather than free (unbound) C5, and clinicians should be aware of this distinction when considering trial-based monitoring thresholds in routine practice. Drug level monitoring is discussed further in the Assessing Adequacy of Complement Blockade section below.

CH50 suppression to near-zero confirms terminal pathway blockade for both agents, although depending on the assay methodology, CH50 is less reliably suppressed with ravulizumab (Willrich et al., 2021b). For ravulizumab, intercurrent illness warrants vigilance given that increased complement activation can lower effective drug adequacy between its extended dosing intervals.

For anti-Factor H antibody-associated aHUS receiving immunosuppression, anti-Factor H titers should be monitored serially; sustained reduction guides decisions about immunosuppression duration and, in selected patients, complement inhibitor discontinuation.

Treatment discontinuation requires close monitoring and access to immediate treatment resumption for recurrence, as relapse risk varies substantially by genotype (Gurevich and Landau, 2023). Weekly to biweekly assessment is recommended initially, with heightened vigilance during intercurrent illness.

#### Transplant-associated TMA

sC5b-9 is the central biomarker for TA-TMA monitoring, with diagnostic and therapeutic response criteria established by international consensus (Schoettler et al., 2023; Schoettler et al., 2025a). Diagnosis requires evidence of microangiopathic hemolytic anemia (schistocytes, elevated LDH, low haptoglobin), thrombocytopenia, and end-organ dysfunction; sC5b-9 at or above 244 ng/mL confirms complement activation and defines the threshold for high-risk disease classification.

Risk stratification is biomarker driven. Standard-risk TA-TMA, defined as elevated sC5b-9 without proteinuria or multiorgan dysfunction, warrants twice-weekly surveillance and supportive measures including calcineurin inhibitor modification. High-risk TA-TMA is defined by proteinuria (protein-to-creatinine ratio at or above 1 mg/mg) combined with elevated sC5b-9, or either lab finding with multiorgan dysfunction. As 1-year non-relapse mortality exceeds 80% in high-risk TA-TMA without complement-directed therapy (Schoettler et al., 2023), prompt complement inhibition is warranted.

Complete response requires sC5b-9 normalization below 244 ng/mL and protein-to-creatinine ratio below 0.2 mg/mg. Incomplete response or persistent sC5b-9 elevation warrants continued or escalated therapy. In patients receiving eculizumab, elevated sC5b-9 increases drug target burden, accelerating drug consumption and frequently requiring doses above standard protocols to maintain suppressed CH50 levels (Jodele et al., 2020). Integrating sC5b-9 levels, eculizumab trough concentrations, and CH50 levels into a precision dosing strategy has been associated with improved outcomes in pediatric TA-TMA.

Monitoring differs for narsoplimab (Matsui et al., 2026). As a lectin pathway-selective inhibitor, it does not suppress CH50 or AH50; monitoring focuses on TMA response markers (hematologic parameters, renal function, and sC5b-9 normalization) rather than complement blockade adequacy.

#### Paroxysmal nocturnal hemoglobinuria

PNH monitoring centers on hemolysis markers. LDH is the most sensitive intravascular hemolysis marker; C5 inhibitors produce rapid, sustained LDH reduction to normal or near-normal levels (Gavriilaki et al., 2022). Additional parameters include hemoglobin, reticulocyte count, haptoglobin, indirect bilirubin, and transfusion requirements.

C5 inhibition does not prevent proximal complement activation, and C3 fragments continue depositing on PNH erythrocytes. Approximately 20%–25% of patients develop clinically significant extravascular hemolysis, characterized by a positive direct antiglobulin test for C3d, persistent reticulocytosis despite normalized LDH, and ongoing anemia with low haptoglobin not explained by intravascular hemolysis (Duval et al., 2026). This pattern identifies patients who may benefit from proximal complement inhibitors targeting C3, Factor B, or Factor D as add-on therapy to C5 inhibition.

Breakthrough hemolysis is defined as LDH more than 1.5 times the upper limit of normal with a hemoglobin drop of 2 g/dL or greater, or with hemoglobinuria, fatigue, or abdominal pain. In phase 3 trials, 11%–27% of patients on standard eculizumab experienced breakthrough hemolysis, often associated with infections, surgery, or infusion intervals exceeding 17 days (Gavriilaki et al., 2022). Symptom recurrence before a scheduled infusion warrants prompt LDH assessment and consideration of dose intensification. PNH clone size should be monitored periodically, typically annually.

#### C3 glomerulopathy

No single biomarker reliably reflects disease activity in C3G, and complement levels correlate poorly with treatment response (Vivarelli et al., 2022). Quantitative proteinuria is the most clinically relevant response marker; in the APPEAR-C3G and VALIANT trials, sustained responses included increased mean C3 and decreased sC5b-9, though proteinuria reduction remains the primary endpoint guiding management decisions (Kavanagh et al., 2025; Fakhouri et al., 2025). Monitoring every 1–3 months during active treatment is appropriate; eGFR should be followed longitudinally, as changes may be gradual. In C3G patients receiving eculizumab, persistently elevated C3d and low C3 despite treatment indicate ongoing complement activation at the C3 level that terminal pathway inhibition cannot address (Wehling et al., 2017). Treatment with pegcetacoplan may increase serum C3 levels despite effective complement inhibition due to accumulation of drug-bound (and inactive) C3 remaining detectable by standard assays (Kolev et al., 2023); such an increase of C3 may serve as confirmation of adherence to the therapy, and C3 activation fragments or functional assays are better indicators of treatment adequacy. C3NeF titers should be monitored serially when present at baseline, as relapsing patients are more often C3NeF positive (Vivarelli et al., 2022). Repeat kidney biopsy may be appropriate when clinical parameters of treatment response are ambiguous or to assess histologic response in the context of clinical trials.

#### IgA nephropathy

Monitoring for IgAN patients receiving complement-targeted therapy focuses on proteinuria reduction (Antonucci et al., 2024). Protein-to-creatinine ratio reduction of ≥50% represents a significant response; eGFR, serum C3, blood pressure, and renin-angiotensin system blockade require ongoing monitoring. Pediatric-specific monitoring protocols are evolving, and adult-derived thresholds should be applied with caution in younger patients.

### Assessing adequacy of complement blockade

#### Drug level monitoring

Therapeutic drug monitoring is not universally standardized but has established utility in specific contexts (Gavriilaki and Brodsky, 2020; Mohebnasab et al., 2019). In PNH, eculizumab trough levels must remain above 35 μg/mL for hemolysis control; trough levels of 50–100 μg/mL are recommended for aHUS (Gurevich and Landau, 2023). In TA-TMA, variable eculizumab clearance requires pharmacodynamic monitoring; target levels above 99 μg/mL were associated with pediatric TA-TMA response, and children frequently required higher doses than standard adult protocols. Patients with higher sC5b-9 have more circulating drug targets and may require more intensive dosing (Wehling et al., 2017). Ravulizumab’s extended half-life provides more consistent trough levels (with a trough target goal of >175 μg/mL); breakthrough hemolysis was lower versus eculizumab in phase 3 trials (4.0% vs. 10.7%) (Gavriilaki et al., 2022).

#### C5 monitoring considerations

Free (unbound) C5 measurement assesses blockade adequacy in C5 inhibitor recipients (Ricklin et al., 2017). Complete blockade produces undetectable free C5 (<0.5 μg/mL); detectable levels suggest incomplete inhibition. Free C5 assays are available through specialized laboratories, though access is limited.

A small subset of patients demonstrates C5 polymorphisms affecting drug binding; the p.Arg885His variant (c.2654G>A), present in approximately 3% of Japanese individuals but rare in most other populations, markedly reduces eculizumab and ravulizumab binding (Bouwman and Guchelaar, 2024). Crovalimab, which binds a distinct epitope on the C5 beta chain, is unaffected (Gavriilaki and Brodsky, 2020). Genetic testing for C5 variants should be considered in patients with poor response despite adequate dosing and confirmed compliance, particularly in populations where these polymorphisms are more prevalent.

#### CH50 and AH50 suppression

Persistent measurable CH50 with terminal pathway inhibition suggests incomplete blockade; several centers use CH50 below 10% as treatment targets. In TA-TMA, pediatric patients often require higher doses or shorter dosing intervals than standard regimens to achieve and maintain this threshold. AH50 should similarly be suppressed.

##### sC5b-9 during treatment

With C5 inhibitors, sC5b-9 should be suppressed, as these agents prevent C5 cleavage and MAC assembly (Ricklin et al., 2017). Elevated sC5b-9 despite therapy suggests inadequate blockade. In TA-TMA, integrating sC5b-9 monitoring with eculizumab levels into a precision dosing strategy has been associated with improved outcomes (Jodele et al., 2024). For proximal complement inhibitors targeting C3 or Factor B, sC5b-9 suppression indicates effective terminal pathway blockade achieved through upstream inhibition (Kolev et al., 2023), as seen with pegcetacoplan in the VALIANT trial and iptacopan in the APPEAR-C3G trial (Kavanagh et al., 2025; Fakhouri et al., 2025).

#### Monitoring frequency

Monitoring frequency should be individualized based on disease severity, treatment phase, genotype, and clinical stability (Antonucci et al., 2024). During the acute phase, assessment is daily to weekly; during early maintenance, weekly to monthly for 6 months with attention to hematologic parameters, renal function, and symptom recurrence; once stable, every 2–3 months for at least 2 years (CBC, C3, creatinine, urinalysis, protein-to-creatinine ratio). Intercurrent infections, surgery, pregnancy, or other complement-amplifying stressors warrant increased monitoring regardless of treatment phase.

#### Monitoring for treatment complications

Given the increased risk of invasive encapsulated bacterial infections during complement inhibitor therapy, any febrile illness warrants prompt assessment, and vaccination status should be verified at each visit (Garred et al., 2021). For patients receiving avacopan and danicopan, liver function tests (AST, ALT, bilirubin) should be monitored at baseline and periodically given the hepatotoxicity risk identified in clinical trials. Anti-drug antibodies can develop against monoclonal antibody and peptide complement inhibitors and may affect long-term efficacy; routine immunogenicity testing is not standard practice but should be considered in patients with unexplained loss of response despite adequate dosing and confirmed compliance.

## Section 4: critical gaps in diagnosis, monitoring, and treatment

### Introduction

The preceding chapters have established the available therapeutics and laboratory framework supporting complement-mediated disease management. These advances have illuminated new challenges: the need for earlier and more precise diagnosis, opportunities to optimize monitoring practices, and the need for greater inclusion of pediatric populations in clinical research. This chapter examines those challenges as opportunities for the next phase of progress.

### Diagnostic gaps

#### Delays in disease recognition

Complement-mediated diseases frequently present as mimics of more common conditions, narrowing the diagnostic window for maximally effective complement inhibition and prevention of permanent injury. Atypical HUS can be clinically indistinguishable from STEC-HUS at presentation, and up to 25% of STEC-HUS cases lack the expected diarrheal prodrome, compounding misclassification risk. The optimal window for complement inhibitor initiation in aHUS (ideally within 24–48 h of presentation) is frequently missed, resulting in preventable and sometimes irreversible renal injury. C3 glomerulopathy presents a parallel challenge: it closely resembles PIGN at initial evaluation, and current consensus recommends observing for C3 normalization over 8–12 weeks after diagnosis before pursuing biopsy and complement evaluation. While this practice avoids unnecessary testing in self-limited PIGN, it creates a diagnostic interval during which progressive complement-mediated injury may occur. Anti-Factor B antibodies may represent a potential discriminatory biomarker, as they were detected in 91% (31/34) of children with PIGN, versus 14% (4/28) with C3G (Chauvet et al., 2020), although further validation is necessary before clinical application of these findings. Rapid genetic screening and point-of-care complement testing to distinguish aHUS from STEC-HUS or PIGN from C3G could provide significant clinical benefit in reducing preventable and permanent injury.

#### Limited availability of specialized testing

Routine complement tests (serum C3, C4, and CH50) are available in most clinical laboratories. The tests most relevant to complement-mediated disease management are not. Soluble C5b-9, C3 nephritic factor assays, free C5, anti-Factor H antibodies, and pathway-specific functional assays require specialized reference laboratories, with turnaround times measured in days to weeks rather than hours. Geographic and global disparities compound this limitation: many pediatric centers worldwide, particularly those outside major academic medical systems, lack direct access to comprehensive complement diagnostic panels.

#### Genetic testing limitations

Genetic evaluation plays a significant role in aHUS management, guiding treatment duration decisions, informing transplant risk stratification, and directing family counseling. Variants of uncertain significance are common, creating interpretive uncertainty that complicates clinical decision-making. The *CFH*/*CFHR* genomic region poses particular challenges: copy number variations, rearrangements, and hybrid alleles in this region are not reliably detected by standard sequencing and require multiplex ligation-dependent probe amplification (MLPA). Genetic testing identifies a causative mutation in only approximately 60% of aHUS cases and 20%–30% of C3G cases, leaving substantial proportions without a defined molecular etiology (Stolbova et al., 2020; Bu et al., 2016). Incomplete penetrance further complicates counseling, as many individuals harboring pathogenic variants never develop disease. Furthermore, the complotype, defined as the inherited constellation of common functional variants across complement genes, represents a critical modifier of disease expression; individually modest effects on complement activation collectively produce substantial and clinically meaningful alterations in pathway activity (Harris et al., 2012). This potentially accounts for a significant proportion of variable clinical phenotypes observed among individuals carrying the same pathogenic variant.

#### Lack of validated biomarkers

The field currently lacks validated biomarkers that reliably reflect disease activity, predict treatment response, or determine therapy duration. In C3G, serum C3 correlates poorly with disease activity, and no established biomarker predicts response to complement inhibition. Urinary complement markers, including urinary C3d and sC5b-9, show promise as indicators of intrarenal complement activation but lack prospective clinical validation. In young children, allowable sample volumes limit the complement assay panels that can be obtained safely, and age-specific ranges for complement components and activation markers remain poorly defined. The etiologic heterogeneity underlying C3G pathophysiology suggests different patients may require fundamentally different monitoring approaches, yet the tools to guide individualization are not available.

Complement disease diagnosis represents a similar testing gap that could benefit greatly from biomarker advancements. aHUS remains a diagnosis of exclusion that is dependent on serologic and genetic evaluations, which can take days to weeks for definitive results. Rapid functional assays assessing complement activation on endothelial cell surfaces have been investigated but have not undergone rigorous validation, and these are addressed later in this review.

Assays evaluating Factor H, Factor I, and MCP functional capacity would identify defects missed by quantitative protein measurement, as some mutations impair function without reducing serum levels or surface expression. In C3G, identifying prospectively which patients will benefit from terminal versus proximal pathway inhibition represents an unmet need. In aHUS, pre-treatment biomarker signatures predicting complete versus partial response to complement inhibition would meaningfully inform therapeutic approach.

#### Recognizing extravascular hemolysis in PNH under C5 inhibition

A diagnostically important gap in PNH management is failing to recognize extravascular hemolysis. With effective C5 inhibitor therapy, intravascular markers (LDH, hemoglobinuria) normalize, but C3 fragment-mediated destruction may perpetuate anemia, risking misattribution to residual disease rather than a second complement-mediated mechanism. C3d-positive DAT with persistent reticulocytosis despite normalized LDH should prompt flow cytometry for C3d-opsonized erythrocytes, identifying patients who may benefit from escalation to proximal complement inhibition (Duval et al., 2026).

### Monitoring gaps

#### Standardization of monitoring protocols

Expert consensus recommendations for complement monitoring exist but are not uniformly implemented, and protocols vary substantially across centers. There is no universally accepted standard for monitoring frequency, the specific parameters to assess at each interval, or the thresholds that should prompt therapeutic intervention. Core questions remain unanswered: optimal monitoring intervals during stable disease, thresholds warranting clinical action versus observation, and the role of repeat biopsy in routine surveillance.

#### Assessing adequacy of complement blockade

Standardized, clinically accessible assays confirming adequate complement blockade during therapy are an unmet need. For C5 inhibitors, CH50 suppression to near-zero provides a functional index of terminal pathway blockade, but free C5 assays offering more direct assessment are not widely available. For proximal inhibitors targeting C3 or Factor B, the optimal parameters for confirming adequate blockade, including the thresholds that predict clinical response, are less well defined.

#### Distinguishing active disease from chronic damage

The inability to reliably distinguish ongoing complement-mediated injury from established chronic damage represents a monitoring gap with direct therapeutic consequences. Persistent proteinuria in C3G may reflect active glomerular inflammation requiring treatment intensification, or it may reflect irreversible scarring unlikely to respond to additional complement blockade. Non-invasive strategies, including urinary biomarker panels combining podocyte markers, tubular injury markers, and complement activation products, as well as emerging biomarkers (such as complement-bound extracellular vesicles), could reduce dependence on repeat biopsy, but none has been prospectively validated for this purpose.

#### Long-term safety surveillance

Many children with complement-mediated diseases will require therapy measured in decades, yet long-term safety data for chronic complement inhibition in pediatric populations remain limited. Beyond infection risk from encapsulated organisms, the cumulative consequences of prolonged suppression on immune maturation, novel pathogen response, and vaccine immunogenicity over a lifetime of treatment are poorly characterized. The established structure of clinical trials required for drug approval is inherently unable to capture the developmental consequences of complement inhibition initiated in early childhood. Post-marketing registries and long-term follow-up programs are essential but remain incompletely enrolled.

### Treatment gaps

#### Limited pediatric-specific trial data

**FIGURE 4:**
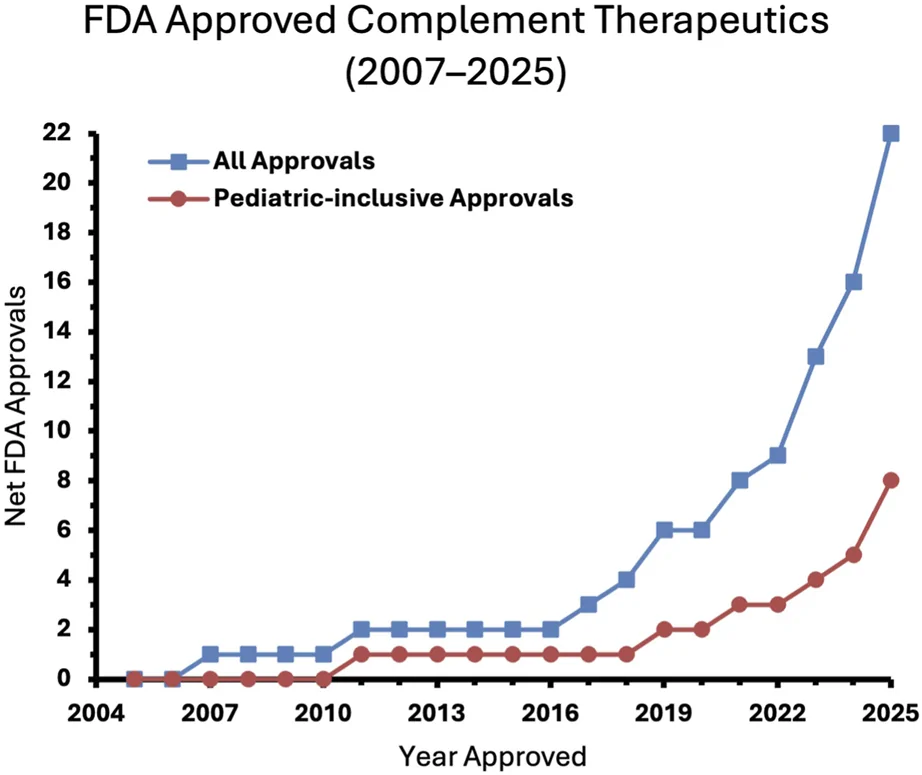
FDA-approved complement-targeted therapeutics, 2007–2025: cumulative approvals by year. Cumulative FDA approvals for complement-targeted therapeutics from 2007 through 2025 are shown, stratified by all approvals (blue) and pediatric-inclusive approvals (red). Total approvals reached 22 by the end of 2025, with marked acceleration from 2021 onward. Pediatric-inclusive approvals reached 8 by the end of 2025, illustrating the persistent gap between drug availability for adults and children across this therapeutic class. FDA withdrawal proceedings for avacopan were initiated in 2026, after the period shown, and were not decided at the time of publication.

**FIGURE 5:**
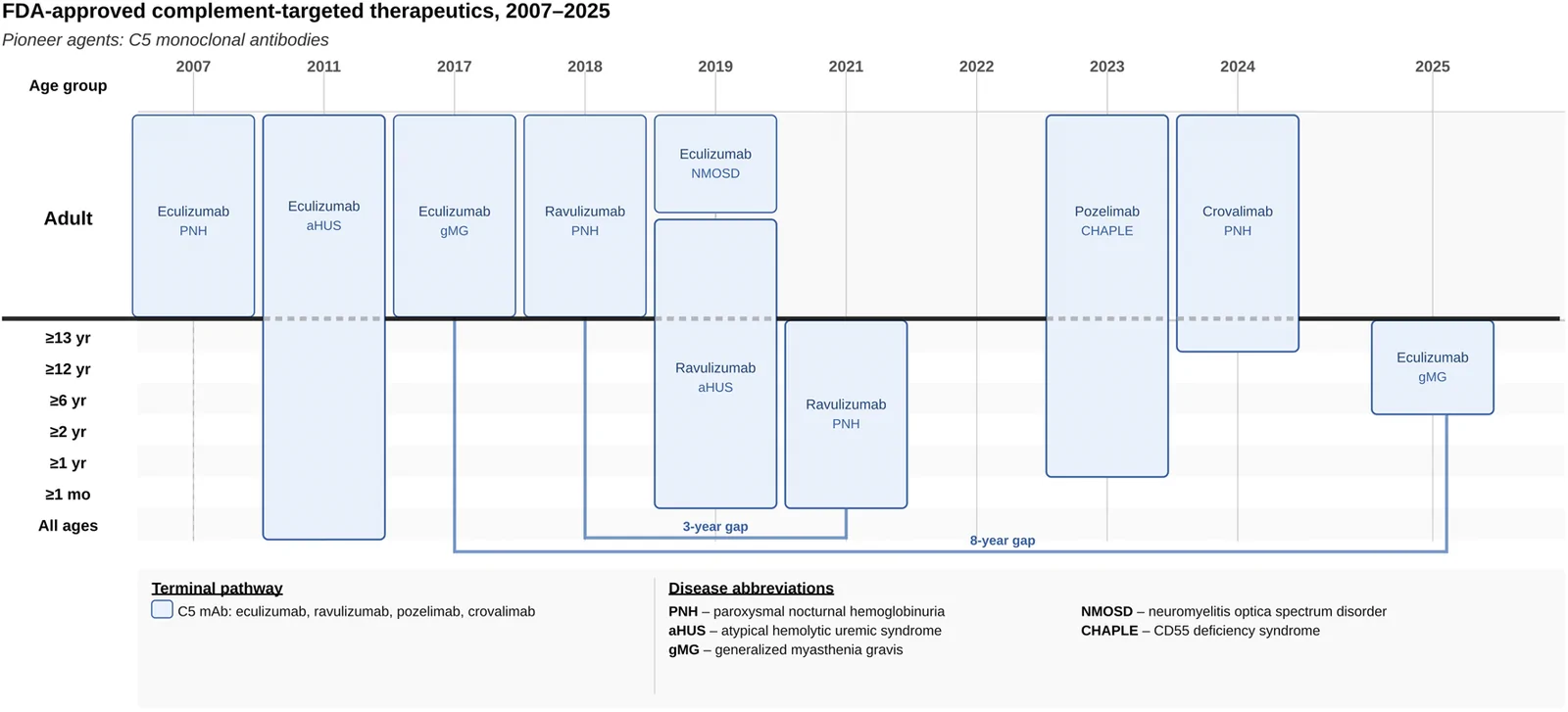
The 3-year and 8-year gaps reflect the intervals between adult and pediatric approval for PNH (ravulizumab, 2018-2021) and gMG (eculizumab, 2017-2025), respectively; notably, the aHUS approvals of both eculizumab (2011) and ravulizumab (2019) included pediatric patients from the outset.

**FIGURE 6:**
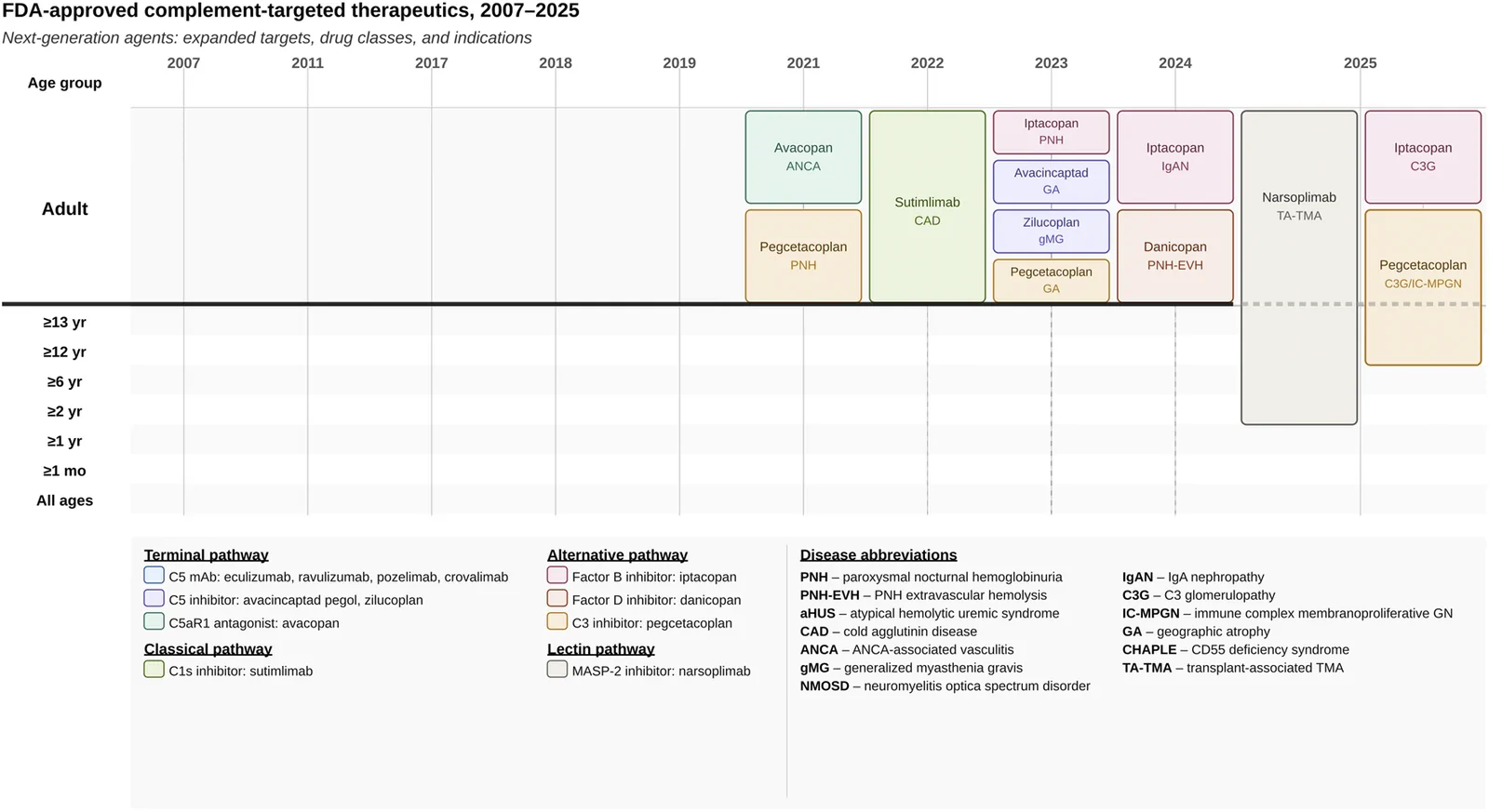
FDA approval timeline for next-generation complement agents (expanded targets, drug classes, and indications), 2007–2025. Approval timeline for non-C5-antibody complement therapeutics is shown by age group and indication from 2007 through 2025. Pediatric-inclusive approvals in this class were absent through 2024, highlighting the ongoing lag in extending newer complement-targeted agents to younger populations. ANCA, antineutrophil cytoplasmic antibody-associated vasculitis; CAD, cold agglutinin disease; gMG, generalized myasthenia gravis; GA, geographic atrophy; IgAN, IgA nephropathy; C3G, C3 glomerulopathy; IC-MPGN, immune complex membranoproliferative glomerulonephritis; TA-TMA, transplant-associated thrombotic microangiopathy; PNH-EVH, PNH with extravascular hemolysis. Note: the pivotal trial supporting the 2021 avacopan approval was retracted in June 2026, and regulatory review of the FDA approval is ongoing.

Complement inhibitors, like many therapeutics in pediatric medicine, reach younger patients primarily through extrapolation from adult trials (Figures 4–6). Off-label and unlicensed drug use affects 36%–97% of hospitalized children across therapeutic categories, reflecting the broader challenge of evidence generation in pediatric populations (Moulis et al., 2018). Pharmacokinetic differences, including faster drug clearance, higher complement turnover rates, and developmental changes in complement component levels, make simple dose extrapolation from adults unreliable. The inconsistent presence of prospective pediatric pharmacokinetic and pharmacodynamic data means early patients in each new drug class have often received empirically estimated dosing whose adequacy is confirmed, if at all, only through therapeutic monitoring or clinical outcome.

#### Incomplete response and treatment-refractory disease

Not all patients achieve adequate responses. In aHUS, some patients have persistent microangiopathic activity or progressive kidney dysfunction despite complement blockade, raising questions about whether inhibition is adequate, whether the diagnosis is correct, or whether pathophysiologic mechanisms outside the complement pathway are contributing. In C3G, treatment response to complement inhibition is less predictable than in aHUS, and this pathophysiologic heterogeneity likely drives differential responses that cannot currently be stratified.

#### Optimal treatment duration

Treatment duration remains among the most consequential unanswered questions in complement therapeutics. The SETS aHUS trial demonstrated that eculizumab discontinuation may be feasible in selected patients, with only 3.6% of patients meeting the primary harm outcome and 14.3% experiencing relapse; relapse occurred only in participants with an identified cause of complement dysregulation (Bryant et al., 2025). Genotype-based stratification provides directional guidance: mutations in circulating complement proteins (CFH, CFI, C3, CFB) carry relapse risk exceeding 50% after discontinuation, while MCP mutation carriers have lower risk because MCP-associated disease is primarily cell-autonomous (Gurevich and Landau, 2023; Sahutoglu et al., 2016). However, these population-level probabilities do not enable reliable individual risk prediction, and the clinical and biomarker signatures that would identify safe discontinuation candidates remain undefined.

#### Kidney transplant risk stratification and management

Stratification of recurrence risk in complement-mediated kidney disease after kidney transplantation by genotype is established, as detailed in preceding chapters. What remains unresolved is the optimal prophylaxis protocol. Initiation timing, dosing frequency, duration of post-transplant complement inhibition, and criteria for eventual discontinuation are without consensus. The threshold for pre-emptive versus reactive treatment in intermediate-risk genotypes and the management of patients who relapse post-transplant are not well defined by controlled data (Michael et al., 2022; Gurevich and Landau, 2023).

#### Autoimmune aHUS

Anti-Factor H autoantibody-positive aHUS (DEAP-HUS) is benefitted by immunosuppression in addition to complement inhibition, with plasma exchange as a bridge and rituximab or cyclophosphamide as the preferred immunosuppressive agents; serial anti-Factor H titers guide tapering. This approach is clinically rational but has not been evaluated in controlled trials, and protocols vary substantially across centers with respect to immunosuppressant choice and dosing, titer thresholds guiding tapering, and criteria for defining remission.

#### Access and cost

Annual drug costs exceeding several hundred thousand dollars per patient create access barriers and global disparity, challenges which are inconsistently mitigated by existing assistance programs. Prior authorization delays, coverage denials, and formulary restrictions interrupt treatment and impose clinical risk, falling disproportionately on underserved populations and regions with limited healthcare infrastructure. Biosimilar development may eventually expand access, but cost remains a substantial global barrier to equitable implementation.

#### Limited options for specific conditions

Complement-targeted therapies for several pediatric conditions remain investigational despite meaningful evidence of complement involvement. Pediatric lupus nephritis, IgA nephropathy, and ANCA-associated vasculitis are among conditions where approved complement inhibitors exist for adults, but pediatric indications and data remain limited or absent. Management of rare complement disorders relies heavily on case reports and expert opinion rather than trial data. Notably, the approval of pozelimab for CHAPLE syndrome demonstrates that mechanism-driven drug development in rare pediatric complement disorders is achievable and provides a framework for advancing therapies in other underserved conditions (Ozen et al., 2024).

### Challenges in pediatric complement research and drug development

#### Small patient populations and trial feasibility

aHUS has an estimated incidence of approximately 2 per million children annually; individual academic centers may see only one or two cases per year (Zagozdzon et al., 2024), and C3G is similarly rare. Multi-center international collaboration is required even for basic trial feasibility, and geographic dispersion imposes enrollment challenges that are difficult to overcome. Ethical constraints present an additional challenge: placebo-controlled designs are difficult to justify when withholding treatment carries meaningful risk of permanent organ injury, and while open-label or single-arm designs may be more feasible, they provide a lower standard of evidence than randomized controlled trials.

#### Inadequate trial infrastructure

Complement inhibitors have generally been developed with adults as the primary population, with pediatric studies planned as secondary programs when conducted at all. Financial incentives for rare pediatric disease development, while improved by regulatory mechanisms such as the Pediatric Research Equity Act and orphan drug designation (Brown, 2024), remain insufficient to drive robust industry investment in conditions affecting hundreds rather than thousands of patients. Academic investigator-initiated trials face resource constraints including the unavailability of complement biomarker assays at many pediatric centers, requiring sample shipping that introduces preanalytical variability. Adult-derived endpoints, including eGFR trajectory and proteinuria, may not capture outcomes most meaningful to children and families. Addressing these constraints requires international collaborative networks with age-specific assay standards and patient-centered outcome measures.

### Gaps in understanding disease mechanisms

#### Heterogeneity within disease categories

Current diagnostic categories encompass pathophysiologically heterogeneous populations. C3G illustrates this most clearly: its two histologic subtypes, DDD and C3GN, share a diagnosis yet differ in ultrastructural pattern, and both encompass multiple pathogenic drivers (genetic mutations, C3 nephritic factors, and anti-complement antibodies, among others) that likely confer distinct treatment responses. Treating this complexity as a single entity in clinical trials plausibly explains the challenges in achieving broadly effective complement-targeted therapy in C3G to date. Similar challenges exist in aHUS, where gain-of-function mutations (*C3*, *CFB*) or loss-of-function mutations (*FH*, *FI*, *MCP*) may confer meaningfully different responses to pathway-specific inhibitors, yet tools to rapidly stratify therapeutic approach by mechanism at the time of treatment selection do not yet exist clinically.

#### Tissue-specific and compartmentalized complement activation

In thrombotic microangiopathy, the focal point of complement-mediated injury is the microvascular endothelial surface, and activation may be substantial at this level while systemic complement markers demonstrate either minimal or no change. Circulating complement abnormalities were detectable in only 47%–64% of aHUS patients regardless of disease phase; yet these patient sera induced excessive endothelial C5b-9 deposits *ex vivo*, an effect that normalized in eculizumab-treated aHUS patients despite unchanged circulating complement levels (Noris et al., 2014). A parallel disconnect exists in C3G, where serum C3 reflects systemic alternative pathway consumption but does not reliably index intrarenal complement deposition or glomerular injury (Ahmad and Bomback, 2020). The reliability of serum-based monitoring is therefore currently limited in complement-associated diseases, and validated markers of local complement activity remain an unmet need.

## Summary

The diagnostic, monitoring, and treatment gaps described above represent not only the current boundaries of practice, but the opportunities that will meaningfully advance complement-focused therapies.

## Section 5: emerging therapeutics, diagnostics, and future directions

### Introduction

Prior chapters catalogued the gaps that define the current boundary of complement medicine: diagnostic delays, absent biomarkers, heterogeneous monitoring, limited pediatric trial data, and treatment protocols built largely on observational evidence. This chapter surveys emerging developments in complement therapeutics, diagnostics, and expanding disease indications, with particular attention to pediatric practice.

### Emerging therapeutics

#### Next-generation C5 inhibitors

Cemdisiran is an RNAi therapeutic that suppresses hepatic C5 synthesis rather than blocking circulating protein (Badri et al., 2021). By reducing C5 production at the source, cemdisiran may provide sustained complement suppression with infrequent subcutaneous dosing. Combination strategies pairing cemdisiran with a conventional anti-C5 antibody are being explored on the hypothesis that upstream synthesis suppression combined with downstream protein blockade could achieve more complete and durable inhibition than either approach alone (Devalaraja-Narashimha et al., 2022). Clinical validation of this strategy is ongoing. Nomacopan and zilucoplan are peptide inhibitors of C5 that have been studied in adults with bullous pemphigoid (Sadik et al., 2022) and generalized myasthenia gravis (Howard et al., 2020), respectively, and evaluation of these agents in pediatric patients with complement-mediated diseases is similarly ongoing.

#### Expanding proximal pathway inhibition

The approvals of pegcetacoplan, iptacopan, and danicopan validated proximal pathway inhibition as a clinically effective strategy and created momentum for further development. Danicopan’s approval as add-on therapy in PNH patients with residual extravascular hemolysis despite C5 inhibition demonstrated that alternative pathway blockade at the amplification loop level can rescue persistent anemia (Lee et al., 2023).

ARO-C3, an RNAi therapeutic targeting hepatic C3 synthesis, is under evaluation in complement-mediated renal diseases including C3G and IgA nephropathy in an ongoing phase 1/2a first-in-human study (NCT05083364); early results have been reported but peer-reviewed data are not yet available. If validated in larger trials, C3-targeted RNAi could offer a sustained proximal inhibition strategy with dosing intervals more convenient than existing regimens. The trade-off of near-complete C3 suppression, which abrogates opsonization as a key immune defense function, will require careful long-term safety evaluation, particularly in pediatric patients whose cumulative exposure may span decades.

Vemircopan, a next-generation oral Factor D inhibitor with greater potency and bioavailability than danicopan in preclinical models, has entered clinical evaluation and is under investigation for broader indications including potential monotherapy use (Gadhachanda et al., 2025).

#### Lectin and alternative pathway pipeline

Real-world expanded access program data for narsoplimab in 136 patients demonstrated excellent survival in both children and adults with high-risk TA-TMA, with 1-year overall survival of 75% in pediatric first-line recipients (Schoettler et al., 2025b). Its distinctive safety profile, which requires no meningococcal vaccination, carries no REMS, and has no boxed warning, positions it as a template for lectin pathway-selective approaches beyond TA-TMA. OMS1029, a long-acting MASP-2 inhibitor, is in clinical development. Zaltenibart, a MASP-3 inhibitor targeting alternative pathway activation, completed two phase 2 trials in PNH and entered phase 3 development; phase 2 monotherapy data showed sustained hemoglobin improvement and prevention of both intravascular and extravascular hemolysis in interim analysis (Griffin et al., 2024), with a global phase 3 program in PNH underway.

#### Oral complement inhibitors and formulation advances

Iptacopan’s approval established that effective systemic complement inhibition is achievable with an oral small molecule, and additional oral agents are in development across multiple targets. For pediatric patients requiring long-term therapy, oral formulations represent a meaningful reduction in treatment burden, though each route of administration carries distinct adherence and monitoring considerations. Age-specific pharmacokinetic data are needed before adult dosing can be reliably extended to children, regardless of formulation.

#### Targeted approaches for specific mechanisms

Pathophysiologic heterogeneity within complement-mediated disease categories is driving development of approaches aimed at focused complement modulation rather than broad pathway blockade. Gene therapy approaches, including liver-directed Factor H or Factor I restoration and anti-C5 constructs, hold conceptual appeal for children facing decades of treatment but remain preclinical or early clinical for most applications (Dreismann et al., 2023). Long-term durability, vector immunogenicity, and off-target genomic effects require evaluation before pediatric application is feasible.

### Emerging diagnostic approaches

#### Novel biomarkers

Cell-bound complement activation products, including erythrocyte-bound C4d and C3d, may be more stable than fluid-phase fragments and more sensitive than serum protein levels as indicators of *in vivo* complement activation. Fluid-phase activation fragments, including C3a, C5a, and factor Bb, have been studied as disease activity markers in complement-mediated renal disease, but their routine clinical utility remains limited by measurement and standardization challenges (Frazer-Abel et al., 2016). Factor H-related proteins, particularly FHR-1, FHR-3, and FHR-5, have been investigated as circulating markers of alternative pathway dysregulation in C3G and IgAN, with levels correlating with disease activity in some cohorts (Kaartinen et al., 2019). Urinary complement markers, including urinary C3d, sC5b-9, and factor Bb, may reflect intrarenal complement activation independent of systemic levels and have shown preliminary signal across several forms of autoimmune glomerulonephritis including IgAN (Genest et al., 2022).

A cell-based functional assay using complement-deficient cell lines incubated with patient serum has shown diagnostic potential for distinguishing aHUS from other thrombotic microangiopathies by detecting alternative pathway-mediated cytotoxicity (Gavriilaki et al., 2015). Broader prospective validation across diverse populations and centers will be required before it can be adopted as a standard clinical tool. The endothelial C5b-9 deposition assay, which measures complement activation on cultured endothelial cells using patient serum, offers a functionally relevant readout for conditions such as aHUS and TA-TMA; as standardization and wider deployment progress, its clinical role is expected to grow (Galbusera et al., 2019; Gastoldi et al., 2023; Meuleman et al., 2022).

#### Rapid and point-of-care testing

Point-of-care platforms for complement activation markers capable of same-day or same-shift results are in development, with lateral flow formats among those under evaluation (Kim et al., 2019; Rodriguez de Cordoba et al., 2024). Clinical validation (establishing sensitivity, specificity, and actionable thresholds in prospective cohorts) remains the critical obstacle between development and deployment.

#### Multiomics and clinical decision support

Proteomic and transcriptomic approaches applied to plasma, urine, or biopsy tissue from complement-mediated disease patients are generating candidate biomarker signatures with potential relevance to disease subclassification, treatment response prediction, and relapse risk. Machine learning approaches applied to integrated clinical, laboratory, genetic, and histopathologic datasets hold potential for improving TMA differentiation and predicting treatment response in complement-mediated disease, though prospective validation is lacking. These approaches remain investigational, and for rare diseases with small patient populations, adequate validation cohorts are themselves a limiting factor.

Clinical decision support tools integrating complement testing interpretation with therapeutic guidance could reduce diagnostic delays and support non-specialist management. Development and validation face the same rare disease constraints: limited patient numbers, heterogeneity within disease categories, and expert consensus as the only available reference standard.

### Expanding disease indications

#### IgA nephropathy

The FDA approval of iptacopan for IgAN in adults, initially based on the APPLAUSE-IgAN trial demonstrating proteinuria reduction in adults (Barratt et al., 2026) and confirmed at 24 months to significantly slow kidney function decline in adults (Barratt et al., 2026), represents the most significant recent expansion of complement-targeted therapy beyond PNH and aHUS. Ravulizumab also demonstrated a 30.1% reduction in proteinuria and eGFR stabilization at 26 weeks in a phase 2 trial in adults with IgAN (Lafayette et al., 2025), supporting the ongoing phase 3 I CAN trial in adults (NCT06291376). Dedicated pediatric trials are now underway: APRICOT (NCT06994845) is a phase 3 trial of iptacopan in children ages 2 to under 18 with IgAN, and I CAN Junior (NCT07024563) is a phase 3 trial of ravulizumab in children ages 2 to under 18 with IgAN or IgAVN (IgA Vasculitis with Nephritis) (Kateifides et al., 2025). These represent the first complement inhibitor trials designed specifically for pediatric IgAN.

#### Lupus nephritis

Complement-targeted approaches are under investigation in lupus nephritis, where classical pathway activation by immune complexes drives glomerular injury, but where complement also paradoxically plays a protective role in immune complex clearance and apoptotic debris removal. This dual biology complicates therapeutic targeting. Ravulizumab and iptacopan are each being evaluated in phase 2 trials enrolling adults with proliferative lupus nephritis (NCT04564339, NCT05268289); no results from the lupus nephritis cohorts have been published. The more aggressive course of pediatric-onset lupus nephritis, and the toxicity of current immunosuppressive regimens in growing children, give urgency to developing effective complement-targeted adjuncts in this population.

### Personalized medicine approaches

#### Genotype- and pharmacogenomics-guided therapy

The clearest current example of genetically informed complement inhibitor selection is the C5 p.Arg885His polymorphism, which reduces eculizumab and ravulizumab binding affinity; crovalimab, which binds a distinct epitope on the C5 beta chain, is unaffected (Gavriilaki and Brodsky, 2020). More broadly, genotype-phenotype relationships in complement-mediated disease suggest mechanistic targets for therapy selection: gain-of-function mutations in C3 or Factor B implicate the alternative pathway amplification loop, while MCP mutations, which produce cell-surface regulatory defects, raise questions about the optimal match between systemic inhibition and underlying pathophysiology. These relationships are plausible but unvalidated prospectively, and current practice treats patients by disease category rather than genetic mechanism. Establishing genotype-guided treatment selection will require collaborative international studies with systematic genotyping and granular outcome data, a prospect particularly relevant in pediatrics, where optimizing treatment from initiation rather than adjusting empirically over years is the more consequential objective.

### Pediatric-specific considerations

#### Transition of care

Complement therapeutics have converted several previously fatal or severely morbid conditions into chronic diseases compatible with long survival. Growing numbers of children with aHUS, PNH, C3G, and TA-TMA are surviving to adulthood and requiring seamless transfer of highly specialized care. The risks at transition are well recognized in chronic pediatric disease generally: provider discontinuity, insurance coverage gaps, loss to follow-up, and the shift of self-management responsibility from parents to adolescents and young adults. These risks are particularly consequential in complement medicine, where treatment interruption carries risk of acute disease recurrence. Structured transition programs beginning in early adolescence, systematic transfer of genetic testing and treatment history, and collaborative relationships between pediatric complement specialists and adult counterparts are necessary components of comprehensive care programs.

Reproductive counseling should be incorporated into transition planning for adolescents and young adults on complement inhibitors. Current antibody-based C5 inhibitors are IgG-based and can therefore cross the placenta; available cohort data are reassuring, with no maternal deaths in 75 eculizumab-exposed pregnancies (Kelly et al., 2015) and live births in all 19 ravulizumab-managed pregnancies with no developmental abnormalities at median 16-month infant follow-up (Hochsmann et al., 2026), though prospective controlled data are lacking and a registry (NCT06312644) is ongoing. Pregnancy in complement-mediated disease itself carries independent risks of disease flare, thrombotic complications, and preeclampsia, and treatment discontinuation during pregnancy risks recurrence.

### Implementation challenges and future needs

#### Access and equity

Geographic and economic disparities in access to complement diagnostics and therapeutics will widen as the therapeutic landscape expands unless access is addressed proactively. Temperature-stable formulations, simplified administration, and pragmatic diagnostics in resource-limited settings are development priorities that international health equity requires. Patent expiration and biosimilar entry for eculizumab, and eventually other agents, offer a foreseeable mechanism for cost reduction, though regulatory and distribution barriers in lower resource settings will require independent attention.

#### Education and training

The rarity of complement-mediated diseases means that most pediatric providers will encounter them infrequently and maintaining diagnostic and therapeutic competency across an expanding range of conditions and agents is a recognized challenge in pediatric nephrology and general pediatrics alike. Telemedicine consultation with complement specialists and electronic health record-embedded decision support offer scalable mechanisms for extending expertise to centers managing these patients outside of specialized referral settings.

#### Long-term outcome research

Complement inhibitors have substantially improved survival and organ outcomes in pediatric aHUS, PNH, and TA-TMA, with maturing follow-up data beginning to characterize multi-year trajectories. A consequential emerging benefit is the reduction in reliance on corticosteroids and cytotoxic agents whose toxicity burdens in growing children are well established. Whether targeted complement inhibition can durably replace broader immunosuppression, and what new complications will emerge with prolonged pathway blockade initiated in infancy or childhood, remain unanswered. The long-term phenotype of a child maintained on complement inhibition from early life, encompassing immune competence, cumulative infection burden, vaccine responses, and renal trajectory, has not been characterized. Longitudinal patient registries with systematic biospecimen collection and international data sharing represent powerful mechanisms for answering these questions at the scale that rare pediatric disease requires.

## Discussion

Targeted complement inhibition has transformed the prognosis of several previously devastating pediatric diseases, and the pace of therapeutic development continues to accelerate. The field now faces the more complex challenges of precision therapy selection, equitable global access, pediatric-specific trial infrastructure, and the long-term surveillance that decades of pathway blockade in growing children will require. Meeting these challenges will depend on sustained investment in registries, collaborative networks, and pediatric pharmacology as new complement therapeutics continue to be developed.
